# Benchmark MRCI Study of Halogen Oxides: Potential Energy Surfaces and Spectroscopic Constants for X_2_, XO, and X_2_O (X = F, Cl, Br)

**DOI:** 10.3390/molecules31173035

**Published:** 2026-08-28

**Authors:** Imen Selmi, Mohamed Bejaoui, Jamila Dhiflaoui, Patryk Jasik, Józef E. Sienkiewicz, Hamid Berriche

**Affiliations:** 1 Laboratory of Interfaces and Advanced Materials, Physics Department, Faculty of Science of Monastir, University of Monastir, Monastir 5019, Tunisia; imenselmi281991@gmail.com (I.S.); mohamed.bejaoui@ipeim.rnu.tn (M.B.); dhiflaouijamila@gmail.com (J.D.); 2 Physics Department, Preparatory Institute for Engineering Studies of Monastir, University of Monastir, Monastir 5019, Tunisia; 3 Faculty of Applied Physics and Mathematics, Gdańsk University of Technology, Narutowicza 11/12, 80-233 Gdansk, Poland; patryk.jasik@pg.edu.pl (P.J.); jozef.sienkiewicz@pg.edu.pl (J.E.S.); 4 BioTechMed Center, Gdańsk University of Technology, 80-233 Gdansk, Poland; 5 Center for Space Biomedicine and Radiological Protection, Medical University of Gdansk, 80-233 Gdansk, Poland; 6 Mathematics and Physics Department, School of Arts and Sciences, American University of Ras Al Khaimah, Ras Al-Khaimah P.O. Box 10021, United Arab Emirates

**Keywords:** halogen oxides, potential energy surface, MRCI, RKHS interpolation, spectroscopic constants, atmospheric chemistry

## Abstract

High-level quantum chemical methods (MRCI+Q and MRCI+P) combined with large correlation-consistent basis sets (aug-cc-pVnZ, where *n* = Q, 5, 6), were used to investigate the ground-state potential energy curves (PECs) and surfaces (PESs) of the $X_{2}$, XO, and $X_{2}O$ systems (with X = F, Cl, Br). For the triatomic systems, the potential energy data obtained from MRCI+Q/aug-cc-pVQZ calculations in two different Jacobi representations were interpolated using the reproducing kernel Hilbert space (RKHS) method to construct smooth and accurate analytical representations of the PESs. This work provides a detailed comparison of the bonding characteristics of these molecules with those reported in previous studies. It lays the groundwork for future investigations of their dynamics and reactivity.

## 1. Introduction

The molecules $X_{2}$, XO, and $X_{2}O$ (X=F,Cl and Br) are of considerable interest in atmospheric chemistry because they participate in processes relevant to ozone depletion and tropospheric reactivity [1,2]. These compounds, known for influencing atmospheric composition through radical-mediated reactions, can catalytically decompose ozone, raising environmental and health concerns. The electronic configurations of $F_{2}$, ${Cl}_{2}$, and ${Br}_{2}$ in their ground states correspond to closed-shell molecular-orbital occupations, in which each atom contributes one electron to the bonding σ molecular orbital, resulting in a total of two electrons in the σ bond. In the case of heterogeneous molecules XO (X=F, Cl, and Br), the X atom seeks one additional electron for a complete valence shell, while oxygen requires two electrons for stability, leading to the formation of a covalent bond where X shares one of its unpaired p-electrons with one of oxygen’s p electrons. This interaction gives the molecule XO an open-shell electronic structure, making it a radical species with unpaired electrons and, therefore, highly reactive toward bond formation and electron-transfer processes. Difluoride monoxide $F_{2}O$ is a pale-yellow solid at low temperatures and can exist in the gas phase under appropriate conditions. In addition, dichlorine monoxide ${Cl}_{2}O$ is a yellowish-red gas at room temperature, while dibromin monoxide ${Br}_{2}O$ is a brownish-red gas under ambient conditions. Despite their different physical states, all three compounds are strong oxidizing agents. They are known for their ability to undergo oxidation reactions, readily donating oxygen or accepting electrons in chemical reactions, making them powerful oxidizing agents in various contexts. These molecules possess distinctive bonding patterns involving covalent interactions between halogen and oxygen atoms.

Experimental studies have improved our understanding of the reactivity and properties of these molecules and have highlighted the importance of assessing their roles in atmospheric and environmental chemistry. For instance, Douglas et al. [3] provided a detailed analysis of the absorption spectrum of gaseous chlorine, focusing on the isotopic species Cl235. Using high-resolution spectroscopy with a 10-m concave-grating spectrograph and techniques such as isotopic separation and cooling, the study identified 35 absorption bands and determined precise ground-state constants. Focsa et al. [4] presented a high-resolution study of the $B^{3}{{\Pi_{0}}^{+}}_{u}-X^{1}\sum^{+}g$ system of ${Br}_{2}$ using laser-induced fluorescence (LIF) spectroscopy combined with Fourier transform spectroscopy. However, the multiphoton ionization spectra using a dye laser of ClO and BrO radicals were determined by Duignan et al. [5], revealing electronic states D, E and F for ClO and new vibrational progressions E, F, and G for BrO. In their study, Tamassia et al. [6] provided a thorough analysis of free radicals FO, BrO and IO, focusing on the (2-0) overtone bands and using laser magnetic resonance. In addition, Lin et al. [7] highlighted the potential of chlorine-derived oxidants for degrading organic contaminants, illustrating the broader chemical relevance of halogen oxide species. Cornford et al. [8] presented the electronic structure of $F_{2}O$ and ${Cl}_{2}O$ by measuring their valence ionization potentials using photoelectron spectroscopy and comparing the experimental results with ab initio and approximate LCAO-SCF calculations based on Koopmans’ theorem. Notably, in their paper, Allen et al. [9] navigated through the structural intricacies of bromine oxides, aiming to scrutinize the stretching vibrations of matrix-isolated ${Br}_{2}O$. The research study of Muller et al. [10] endeavors to unravel the intricate structural properties of ${Br}_{2}O$ and OBrO, by conducting spectroscopic analysis in the millimeter-wave region.

Alongside these experimental studies, theoretical studies have explored the properties and interactions of these molecules, shedding light on their behavior in the gas phase and their potential environmental impact through the compounds they form during reactions. In this vein, using extensive multiconfigurational CI methods, Cartwright et al. [11] presented a comprehensive ab initio investigation of the electronic structure of $F_{2}$, analyzing its ground and eleven excited electronic states. Langhoff et al. [12] reported high-accuracy ab initio calculations for the ground states of $N_{2}$, $O_{2}$, and $F_{2}$, emphasizing the critical role of large Gaussian basis sets and MRCI methods in determining spectroscopic constants and dissociation energies. Daria et al. [13] presented a detailed ab initio configuration interaction study of the UV photodissociation of ${Cl}_{2}$, using multireference CI (MRCI) with relativistic effective core potentials and Davidson corrections. Using CASPT2, CASPT3, CCSD(T), and AQCC, Leininger et al. [14] presented a comprehensive ab initio investigation of electron attachment to ${Cl}_{2}$, combining experimental insights with high-level computational methods. The results of Peyerimhoff et al. [15] obtained using the MR-ISCI method, provided accurate potential energy curves, offering valuable insights for UV laser applications, spectroscopic analysis, and understanding dissociation dynamics in ${Cl}_{2}$. Theoretical investigations of the electronic structures of the bromine molecule ${Br}_{2}$, were studied by Abdel-Hafiez et al. [16], using the Complete Active Space Self-Consistent Field (CASSCF) method with a 6-31+G(d,p) basis set. It combines time-dependent quantum dynamics and semi-classical theory to calculate absorption cross-sections, transition probabilities, and spectroscopic constants for different states. Dissociation of homonuclear diatomic halogens ${Cl}_{2}$ and $F_{2}$ via multireference coupled cluster calculations was determined by Chattopadhyaya et al. [17]. Liu et al. [18] reported calculations of potential energy curves for dihalogens ($F_{2}$, ${Cl}_{2}$, and ${Br}_{2}$) using the interacting Density Matrix Functional Theory (i-DMFT) method. Moreover, the distinctive reactivity of dihalogen molecules has been the subject of extensive investigations. Barbosa et al. [19] presented the electronic structure of dihalogen molecules $F_{2}$, ${Br}_{2}$ and ${Cl}_{2}$, focusing on the “charge-shift bonding” effect and providing a detailed analysis of the fluorine molecules’ properties. Visscher et al. [20] have theoretically studied relativistic and correlation effects in dihalogens, including bond lengths, frequencies, and dissociation energies. In addition, ab initio calculations for the interaction of $H_{2}O$ with ${Cl}_{2}$ and ${Br}_{2}$ in ground and excited states were analyzed by Hermández-Lamoneda et al. [21], exploring different bond lengths and employing advanced computational techniques. By exploring how molecular halogens ($F_{2}$, ${Cl}_{2}$, ${Br}_{2}$ and $I_{2}$) alter the electronic structure of graphene oxide (GO), Mersal et al. [22] investigated charge transfer to improve light-harvesting capabilities.

Previous theoretical studies have also provided valuable insights into the bonding and spectroscopy of these radical species. Miller et al. [23] presented highly accurate potential energy curves for the FO free radical by performing a global Dunham fit to extensive high-resolution rotational, vibrational, and fine-structure spectra. Comparison with ab initio calculations shows that only methods incorporating extensive electron correlation, large basis sets, and intrinsic relativistic effects such as open-shell relativistic CCSD(T) can reliably reproduce the experimental data, including fine-structure splitting. The 21 electronic states of the ClO radical were investigated by Wang et al. [24], with a particular focus on weakly bound states and the effects of spin–orbit coupling (SOC) on their spectroscopic properties. Using CASSCF and icMRCI calculations with large correlation-consistent basis sets, including core–valence and scalar relativistic corrections, the authors constructed accurate potential energy curves, which were fitted and then used to solve the ro-vibrational Schrödinger equation. A theoretical investigation was conducted by Seung-Joon Kim et al. [25] about the spectroscopic properties of ClO and its anion ${ClO}^{-}$ using high-level ab initio methods with large diffuse basis sets. The authors optimized molecular geometries, calculated vibrational frequencies, and evaluated electron detachment and dissociation energies. Li et al. [26] investigated the ground electronic states of chlorine monoxide ClO, its cation, and its anion, using high-level MRCI, CASSCF, and coupled cluster calculations with large aug-cc-pVXZ basis sets and complete-basis-set extrapolations. Woon et al. [27] conducted a theoretical study, focusing on bonding in chalcogen halide species (O,S,Se)\(F,Cl,Br), comparing stability and bond energies. A thorough examination of the electronic nonadiabatic transitions in the context of photoelectron spectroscopy of $F_{2}O$ was theoretically conducted by Krishnan et al. [28]. Chau et al. [29] presented potential energy functions for various states of ${{Cl}_{2}O}^{+}$ and ${Cl}_{2}O$, using advanced computational methods that incorporate anharmonic effects through Franck-Condon factors. By employing ab initio CI methods, Palmer et al. [30] examined the valence and Rydberg states of ${Cl}_{2}O$ focusing on UV-VUV absorption and photoionization, using advanced methods including MR-CI with extensive Rydberg basis functions, DFT/B3LYP geometries, and GAMESS-UK implementations. The theoretical research of Toniolo et al. [31] identified eight singlet excited states for ClOOCl and thirteen for ${Cl}_{2}O$ by simulating the photoabsorption spectra, offering a comprehensive understanding of the electronic transitions of these molecules. By analyzing energetically low-lying minima, interaction energies, and vibrational frequencies, Zhou et al. [32] performed calculations using MP_2_ and B3LYP levels of theory. This study also provides valuable insights into the hydrogen bonding interactions between ${Cl}_{2}O$ and the HO radical. The research by Papanastasiou et al. [33] studied the rate coefficients for the gas-phase reaction between O(^3^P) and ${Cl}_{2}O$ over a temperature range of 230 to 357 K and addressed discrepancies found in previous investigations. Grein et al. [34] discusses the calculations of vertical excitations for singlet and triplet states of ${Br}_{2}O$. In conclusion, these observations underscore the significance of comprehending and exploiting the attributes of halogen atoms in the realm of scientific progress.

This study systematically investigates diatomic and triatomic halogen-containing species, including $X_{2}$, XO, and $X_{2}O$ (X=F, Cl, and Br), using a consistent computational framework. Ab initio electronic structure methods provide a rigorous approach for describing these molecular systems by solving the Schrödinger equation within a finite basis set representation [35,36,37,38]. However, such calculations remain computationally demanding, particularly for constructing detailed multidimensional potential energy surfaces of halogen-containing systems. In this work, these methods are employed to characterize the ground state properties and spectroscopic characteristics of the diatomic species and to construct and analyze the potential energy surfaces of the corresponding triatomic systems.

The remainder of this paper is organized as follows. Section 2 describes the electronic-structure calculations and the RKHS interpolation procedure used to construct the triatomic potential energy surfaces. Section 3 presents the spectroscopic results for the diatomic species and the PES analysis for the triatomic systems. Section 4 summarizes the main conclusions.

## 2. Computational Approach

### 2.1. Ab Initio Electronic Structure Calculations

All electronic structure calculations were carried out with the MOLPRO 2010 package [39] within the Born–Oppenheimer approximation. To ensure smooth convergence of the multiconfigurational wave function, Hartree-Fock calculations were initially executed to construct a suitable starting set of molecular orbitals. These orbitals were subsequently optimized within the selected active space using the CASSCF method. The optimized CASSCF wave functions then served as reference states for multireference configuration interaction (MRCI) calculations, which incorporated dynamic electron correlation to accurately describe the electronic states and potential energy surfaces.

The multireference configuration interaction (MRCI) methods account for electron correlation effects by including single and double excitations from multiconfigurational reference wavefunctions, usually obtained from a CASSCF calculation. However, standard MRCI is not size-extensive, leading to an underestimation of the correlation energy, particularly near dissociation. To reduce this error, Davidson (+Q) and Pople (+P) size-consistency corrections are commonly applied. The MRCI+Q correction introduces an approximate quadruple excitation contribution based on the Davidson formula, while the MRCI+P correction applies the Pople correction to compensate for the missing higher-order excitations. These corrections improve dissociation energies and spectroscopic properties by recovering part of the correlation energy missing in the truncated CI expansion.

To investigate the ground electronic states of diatomic systems, we employed large-augmented correlation-consistent basis sets [40]. For the homonuclear diatomic molecules $F_{2}$, ${Cl}_{2}$, and ${Br}_{2}$, in their ^1^$\sum_{g}^{+}$ ground states with D_∞h_ symmetry, the cc-pV5Z, aug-cc-pV5Z, and ECP28MWB_AVTZ (Energy-Consistent Pseudopotential, large core) [41] basis sets were used, respectively. Since all halogens have seven valence electrons, the calculations employed the full-valence active space used by MOLPRO, corresponding to CAS (14, 8). This active space includes the 14 valence electrons arising from the s and p valence shells of the two halogen atoms and distributes them over 8 active molecular orbitals. In the D_2h_ symmetry representation, the active orbitals were distributed as (2, 1, 1, 0, 2, 1, 1, 0).

For the heteronuclear diatomic species FO, ClO, and BrO, which exhibit ^2^Π ground states and possess C_∞v_ symmetry, the cc-pVQZ, aug-cc-pV6Z, and ECP28MWB/cc-pVQZ basis sets were employed, respectively. The same active space size was used for these halogen monoxide radicals, which each have 13 valence electrons. Therefore, the CASSCF calculations employed a CAS (13, 8) active space, consisting of 13 active electrons distributed over 8 active molecular orbitals. The active orbitals were distributed according to the C_2v_ symmetry as (4, 2, 2, 0).

For the diatomic molecules, potential energy curves were computed over internuclear distances from 1.0 to 30.0 Å with a step size of 0.1 Å, providing dense sampling of the bound, near-dissociation, and asymptotic regions. A comparative assessment of several electronic structure approaches led us to adopt MRCI as the best compromise between accuracy and computational cost for the systems considered here.

In the second stage of this work, to systematically map the potential energy surfaces of the $X_{2}O$ (X=F, Cl, Br) systems, the MRCI+Q level of theory in conjunction with aug-cc-pVQZ was performed assuming a $C_{s}$ point group symmetry. The ground singlet state adiabatic potential energy surface of $X_{2}O$ was calculated as the lowest singlet state (S = 0) with ^1^*A*′ irreducible representation in $C_{s}$ point group symmetry using MRCI+Q with CASSCF reference. This state correlates asymptotically to distinct products depending on the dissociation pathway: (i) $X_{2}$ (^1^$\sum_{g}^{+}$)+ $O{(}^{1}D)$ in the $O+X_{2}$ limit, and (ii) XO(Π2)+X(Pu2) in the X+XO limit.

**Figure 1:**
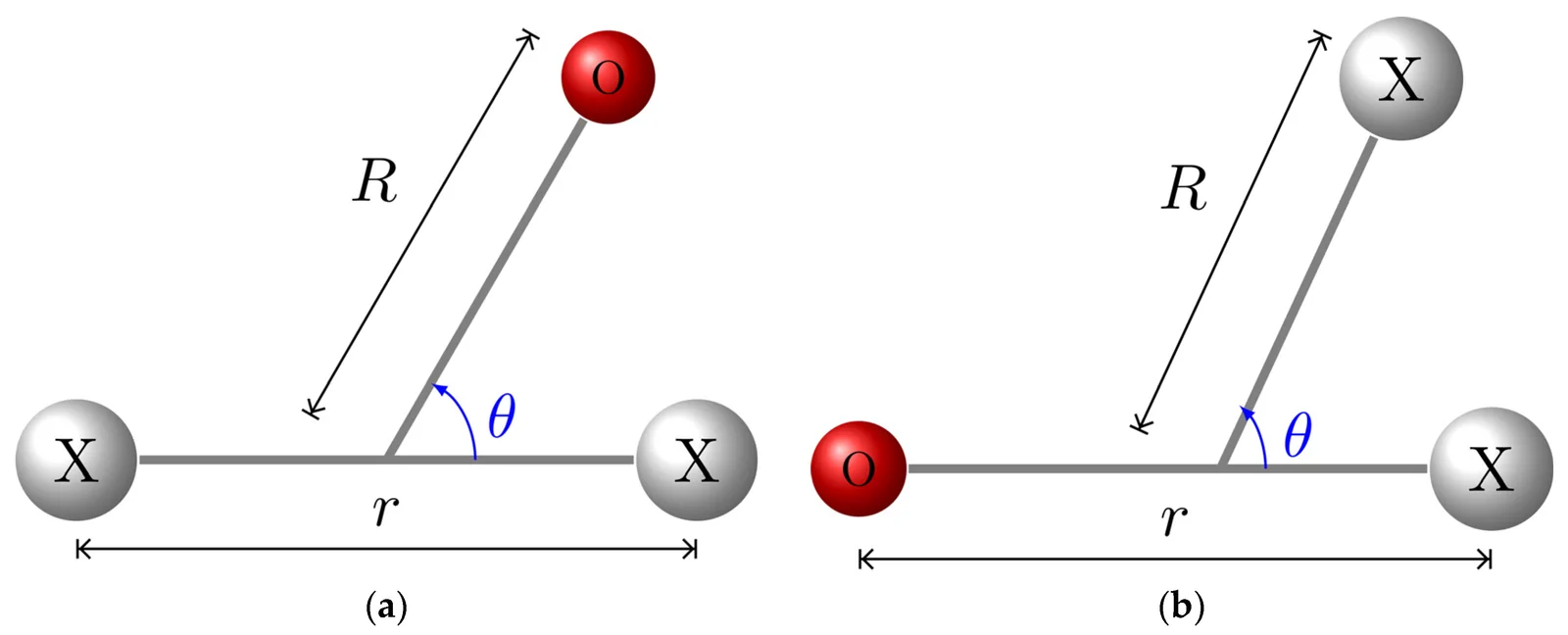
Schematic representation of the two Jacobi coordinate systems used in this work: (**a**) $X_{2}-O$ and (**b**) XO−X.

The MRCI calculations employed full-valence reference spaces. For the X_2_O triatomic systems, 20 valence electrons were treated explicitly as correlated electrons and distributed over 12 valence molecular orbitals, corresponding to a CAS (20, 12) reference space. For Br_2_O, the 78 total electrons comprise 58 frozen core electrons occupying 29 core orbitals and 20 correlated valence electrons. We considered two Jacobi coordinate (r, R, θ) representations. In the first configuration, an O atom approaches a vibrating $X_{2}$ fragment, as illustrated in Figure 1a. In this representation, the X−X bond length (r) is fixed, and *R* denotes the distance from the oxygen atom to the center of mass of the $X_{2}$ fragment. The angle θ is defined as the angle between the vector R and the X−X bond axis, as shown in Figure 1a. For $F_{2}$, ${Cl}_{2}$, and ${Br}_{2}$, the bond lengths were sampled over the ranges 1.6–2.8 Å, 2.2–3.4 Å, and 2.4–3.6 Å, respectively, using a uniform step size of 0.2 Å.

In the second configuration (Figure 1b), the X−O bond was fixed. In this case, R is defined as the distance from the attacking X atom to the center of mass of the XO fragment. θ is now the angle between this vector R and the vector representing the fixed X−O bond axis of the fragment. The X−O bond length was similarly scanned over a range: 1.0–1.8 Å for FO, 1.3–2.1 Å for ClO, and 1.4–2.2 Å for BrO, with a step size of 0.2 Å. These two coordinate representations provide complementary descriptions of the same triatomic PES and allow us to compare the $X_{2}O$ and XO−X arrangements on an equal footing. For the two cases, the angular coordinate θ was varied from 0° to 180°, with the sampling based on a 13-point Legendre polynomial quadrature, ensuring accurate integration over angular dependencies. This approach yielded the following angular grid points: θ = 10.2041°, 23.4225°, 36.7189°, 50.0328°, 63.3530°, 76.6759°, 90.0°, 103.3241°, 116.6470°, 129.9672°, 143.2811°, 156.5775°, and 169.7959°.

Vibrational levels of the diatomic molecules were calculated using the discrete variable representation (DVR) method [42,43]. The resulting vibrational energies were fitted to the Dunham expansion to extract the spectroscopic constants. This procedure yields a consistent and accurate set of spectroscopic parameters from the computed vibrational levels. Standard CODATA constants were used for all unit conversions, and the most abundant isotopes were adopted in the reduced-mass calculations. From the computed curves, we derived the equilibrium bond length $r_{e}$, dissociation energy $D_{e}$, harmonic frequency $\omega_{e}$, anharmonicity constant $\omega_{e}\chi_{e}$, and rotational constant $B_{e}$. The equilibrium rotational constant B_e_ is given by $B_{e}=\frac{\hbar^{2}}{2\mu r_{e}^{2}}$. It is worth noting that for our triatomic molecules, the harmonic frequency $\omega_{e}$ was determined from the second derivative of the potential energy surface at the equilibrium point. Using the triatomic PES, we determined the force constant $K={\frac{\partial^{2}V}{{\partial r}^{2}}|}_{r=r_{e}}$ evaluated at $r=r_{e}$, and used it to compute the harmonic vibrational frequency of the triatomic complex, $\omega_{e}=\sqrt{\frac{K}{\mu }}$, where $\mu^{\prime }$ is the appropriate reduced mass. For the $X_{2}O$ configuration, $\mu =\frac{m_{X_{2}}\times m_{O}}{m_{X_{2}}+m_{O}}$ with $m_{X_{2}}=2m_{X}$. For the XO−X configuration, $\mu =\frac{{(m}_{X}+m_{O})\times m_{X}}{{2\times m}_{X}+m_{O}}$. Note that K is a property of the PES and therefore independent of the atomic masses. For the triatomic systems, the equilibrium geometries for each angular slice (fixed θ) were determined as follows. For each fixed r ($X_{2}O$ configuration) or fixed *X*-*O* distance (*XO*-*X* configuration), the ab initio energy points along the R coordinate were interpolated using cubic splines to construct a continuous potential energy curve. The minimum of each interpolated curve was located using the Broyden–Fletcher–Goldfarb–Shanno (BFGS) optimization algorithm. The stability of each optimized geometry was verified using basin-hopping global optimization, which combines random perturbations with local BFGS optimizations to ensure the global character of the minimum.

### 2.2. Generation of the Potential Energy Surface of Triatomic Complexes

The two-dimensional interaction potential was expanded in a finite series in Legendre polynomials in cosθ, up to λ=12:

(1) $$W\left(R,\theta \right)=\sum_{\lambda =0}^{12}f_{\lambda }(R)P_{\lambda }(cos\theta ),$$

where $P_{\lambda }(cos\theta )$ is the Legendre polynomial of order λ, and $f_{\lambda }(R)$ denotes the radial expansion coefficients, which are commonly referred to as radial strength functions.

To represent the potential energy surfaces analytically, we employed the reproducing kernel Hilbert space (RKHS) interpolation method of Ho and Rabitz [44,45]. For each surface, the interaction energy is expressed as:

(2) $$V(R)=\sum_{K=1}^{M}\alpha_{K}Q(R_{K},R)$$

In this context, $R=(x_{1}^{\prime },\ldots ,\;x_{F}^{\prime })$ denotes the set of internal coordinates specifying a given nuclear configuration at which the interpolated energy is evaluated. The vector $R_{K}=(x_{K1},\ldots ,\;x_{KM})$ corresponds to the (K)-th ab initio data point, and (M) is the total number of such points. The coefficients ($\alpha_{K}$) are determined from the interpolation conditions, while $Q(R_{K},R)$ is the reproducing kernel defined in the RKHS.

The coefficients ($\alpha_{K}$) in Equation (2) are determined by solving the following system of linear equations:

(3) $$\sum_{l=1}^{M}Q_{Kl}\alpha_{l}=V\left(R_{K}\right);\;K=1,\;2,\ldots \ldots ,M$$

where $Q_{Kl}=Q(R_{K},R_{l})$, and $V\left(R_{K}\right)$ denotes the ab initio energy calculated at the (K)-th grid point. Therefore, the multidimensional reproducing kernel used to represent the potential energy surface can be written as a product of one-dimensional kernels:

(4) $$Q(R_{K},R)=\prod_{i=1}^{F}q_{i}\;(x_{Ki},\;x_{i}^{\prime })$$

where $q_{i}(x_{Ki},\;x_{i}^{\prime })$ is the one-dimensional kernel associated with the (*i*)-th internal coordinate. For the one-dimensional distance-like coordinates, the following reciprocal power RKHS kernel was employed:

(5) $$q^{n,m}(x,x^{\prime })=n^{2}\;x_{>}^{-(m+1)}\;B{\left(m+1,n\right)}_{2}F_{1}(-n+1,\;m+1;\;n+m+1;\frac{x_{<}}{x_{>}})$$

where x_>_ and x_<_ are the larger and smaller values of x and x′, respectively. Here, B is the beta function and F12 is the Gauss hypergeometric function. To account for the dominant long-range dispersion interaction between the O atom and the $X_{2}$ dimer, a weighting factor of the form w(x) = x^−m^ with m = 5 was introduced.

In the RKHS scheme, the angular coordinate was transformed into a new variable:

(6) $$z=\frac{1-cos\theta }{2};$$

which maps the angular domain $cos\theta \in \left[-1,\;1\right]$ onto the interval $z\in \left[0,\;1\right]$. This transformation provides a convenient coordinate for constructing an efficient angular grid representation.

We evaluated several kernel forms in order to identify the interpolation scheme that best reproduces the ab initio data. The selection of an appropriate kernel is crucial, as different kernels exhibit distinct behaviors in capturing both short-range and long-range interactions.

In our study, we systematically tested a range of kernels, including reciprocal power, exponential decay, Taylor spline, and Laplacian, with varying parameter settings to assess their impact on interpolation accuracy. Particular attention was given to their ability to reproduce the correct asymptotic behavior of neutral atom-diatom interactions. Among the tested kernels, the reciprocal power kernel provided the highest interpolation accuracy because it naturally reproduces the physically correct long-range interaction proportional to (1/R^6^). Consequently, this kernel was selected for the construction of the final potential energy surface. Additional technical details regarding the RKHS implementation are available in Ref. [46].

RKHS has been effectively utilized to construct PESs for systems such as $NO_{2}$ and $N_{2}O$ [46,47]. We previously generated, in our group, a set of high-accuracy ab initio interaction energies [48]. These ab initio energy points were then used to reproduce an analytical potential energy surface (PES) using the RKHS approach, which is very well adapted to both regular and irregular grids and accommodates critical asymptotic behaviors and molecular symmetry. Our subsequent study on H-LiH collisions [49] demonstrated the importance of the accuracy of PES representations for quantum dynamics applications.

**Figure 2:**
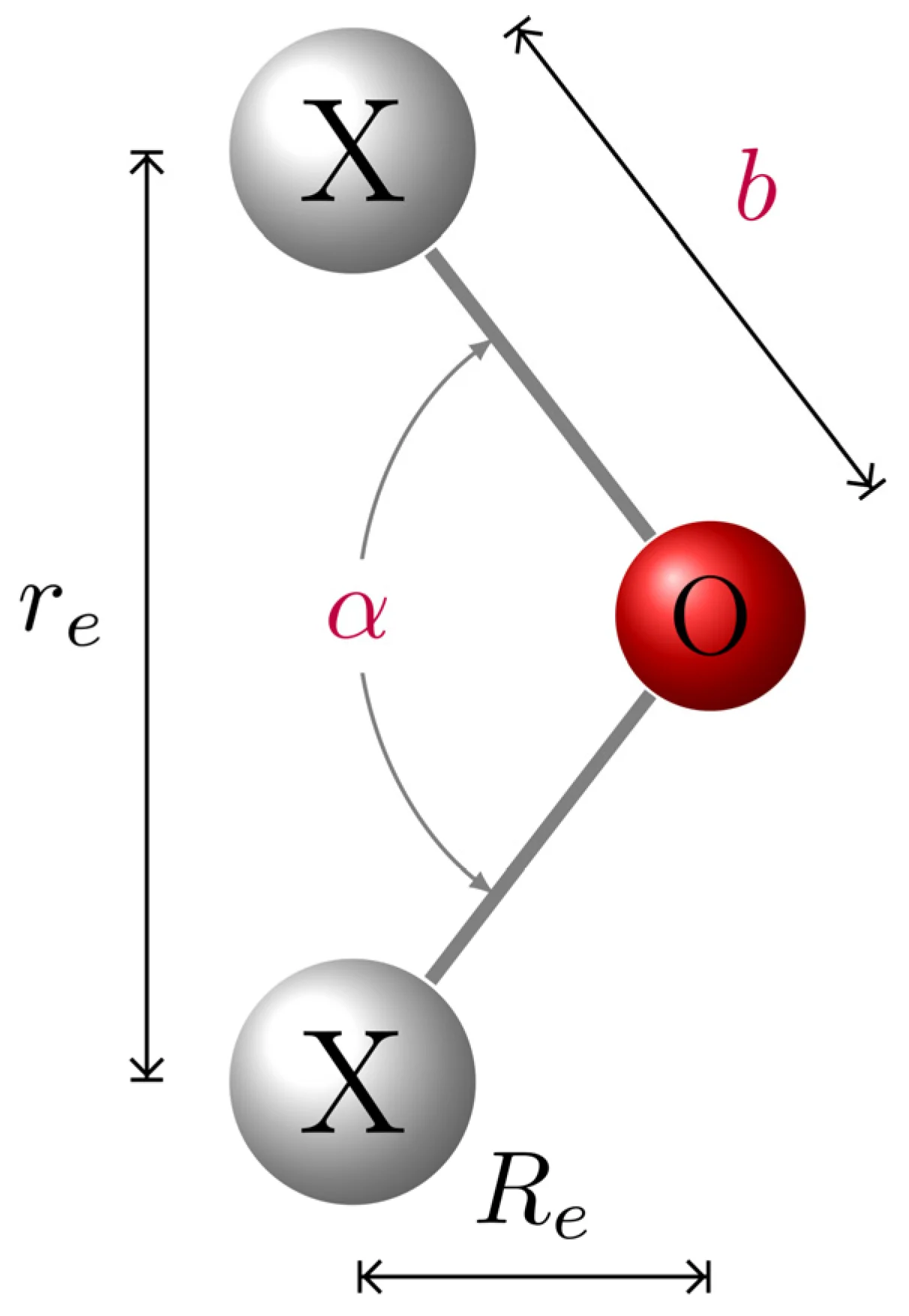
Schematic representation of the equilibrium configuration.

A detailed description of the molecular system in Jacobi coordinates is presented in Figure 2, incorporating key geometric and energetic relationships essential for analyzing the system within the RKHS framework. The individual bond distances between oxygen and each X atom are given by:

$${OX}_{1}=\sqrt{{\left(\frac{r}{2}\right)}^{2}+R^{2}-R\times rcos(\theta )}$$

$${OX}_{2}=\sqrt{{\left(\frac{r}{2}\right)}^{2}+R^{2}+R\times rcos(\theta )}$$

Additionally, at optimized geometry, parameters $r_{e}$ and R are defined as:

$$r_{e}=X_{1}X_{2}=2b\times sin(\frac{\alpha }{2})$$

$$R=b\times cos(\frac{\alpha }{2})$$

The interaction energy from ab initio calculations is given by the difference between the energy of the $X_{2}O$ molecule and the sum of the energies of its constituent atoms:

$$E_{Int}(X_{2}O)=E(X_{2}O)-2\times E(X)-E(O)$$

Using the RKHS interpolation method, the total energy is expressed as the interaction energy from different molecular fragments. For our first Jacobi configuration,

$$E_{Tot}^{RKHS}(X_{2}O)=E_{Int}^{RKHS}\;(X_{2}O)+E_{Int}^{RKHS}(X_{2}(r))+E_{Int}^{RKHS}({OX}_{1})+E_{Int}^{RKHS}({OX}_{2})$$

and for our second Jacobi configuration,

$$E_{Tot}^{RKHS}(XO-X)=E_{Int}^{RKHS}\;(XO-X)+E_{Int}^{RKHS}(X_{1}O(r))+E_{Int}^{RKHS}({X_{1}X}_{2})+E_{Int}^{RKHS}({OX}_{2})$$

where $E_{Int}^{RKHS}\;(X_{2}O)$ and $E_{Int}^{RKHS}\;(XO-X)$ represent the three-body expansion.

## 3. Results and Discussion

### 3.1. Electronic Structure and Spectroscopic Properties of $X_{2}$ and XO Dimers (X = F, Cl, Br)

#### 3.1.1. Homonuclear $X_{2}$ Molecular Systems (X = F, Cl, Br)

**Figure 3:**
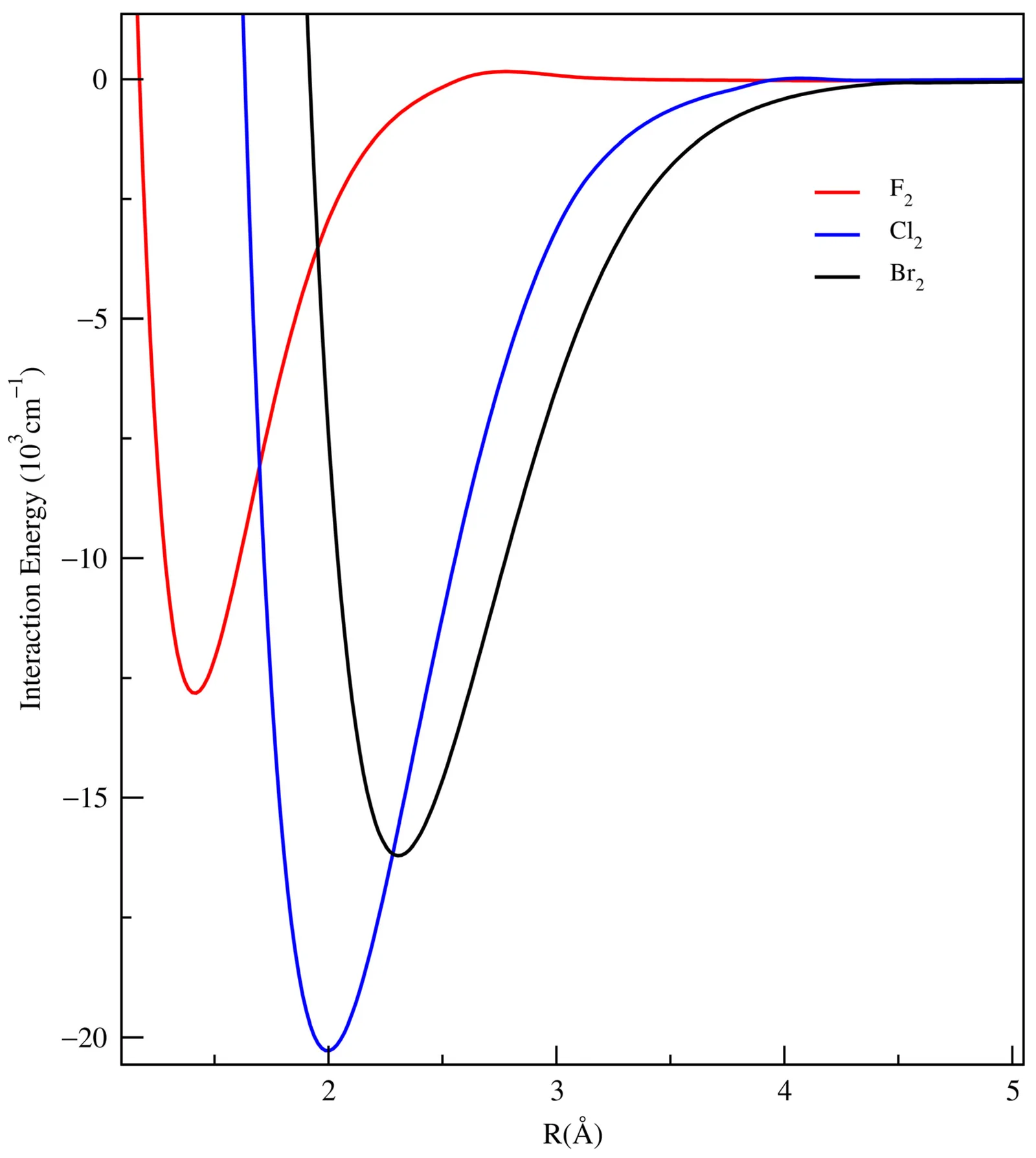
Potential energy curves of the homonuclear diatomic molecules $F_{2}$, ${Cl}_{2}$, and ${Br}_{2}$ in their ground electronic states, computed at the MRCI-based level of theory described in the text.

The potential energy curves (PECs) for the ground electronic states of $F_{2},{Cl}_{2}$ and ${Br}_{2}$ are shown in Figure 3.

Figure 3 shows that the PECs of $F_{2}$, ${Cl}_{2}$, and ${Br}_{2}$ differ substantially in both well depth and equilibrium bond length. The quality of the computed energy surfaces was validated through the calculation of spectroscopic parameters: the equilibrium bond length $r_{e}$, dissociation energy $D_{e}$, harmonic frequency $\omega_{e}$, anharmonicity constant $\omega_{e}\chi_{e}$, and rotational constant $B_{e}$. The spectroscopic constants of the $X_{2}$ molecules, obtained using the MRCI, MRCI+Q, and MRCI+P methods, are presented in Table 1 and compared with available experimental and theoretical results. The calculated spectroscopic parameters show that both corrections improve the agreement with experimental values compared with uncorrected MRCI. The MRCI+Q method generally provides a more accurate description of dissociation energies because the Davidson correction is designed to recover size-extensivity errors in bond-breaking regions. The MRCI+P correction also improves the correlation energy but may show a large dependence on the reference configuration and excitation space.

The derived well depths, denoted as $D_{e}$ are determined based on MRCI+Q calculations to be $12,821.68\;{cm}^{-1}$ for $F_{2},$ $20,249.10\;{cm}^{-1}$ for ${Cl}_{2}$ and $16,204.00\;{cm}^{-1}$ for ${Br}_{2}$ molecules. The reported equilibrium distances for $F_{2},{Cl}_{2}$ and ${Br}_{2}$ are 1.416 Å, 1.997 Å, and 2.306 Å, respectively. Among the halogen diatomics, ${Br}_{2}$ has the longest bond length owing to the large size of bromine atoms, while ${Cl}_{2}$ has the highest dissociation energy. $F_{2}$ is weakened by strong lone pair repulsions, and ${Br}_{2}$ is weakened by its long bond length, leaving ${Cl}_{2}$ with the optimal balance between bond strength and atomic size. Relative to the experimental data of Huber and Herzberg [50], the calculated equilibrium bond lengths for the $X_{2}$ molecules differ by less than 0.3 Å. Additionally, the vibrational frequencies ω_e_ for the $F_{2}$ ${,Cl}_{2}$ and ${Br}_{2}$ molecular systems show deviations of $10\;{cm}^{-1}$, $15\;{cm}^{-1}$, and $18\;{cm}^{-1},$ respectively, from the experimental values [50].

Table 1 shows that the spectroscopic constants of all $X_{2}$ molecular systems, as determined via MRCI+Q and MRCI+P calculations, align with other theoretical works.

Specifically, for $F_{2}$ our electronic structure calculations using the cc−pV5Z basis set are in close accord with the experimental data available [50]; the equilibrium distance $r_{e}$, the dissociation energy $D_{e}$ and the harmonic frequency $\omega_{e}$ present differences of over 0.3%, 3.56%, and 0.6%, respectively, when using MRCI+Q and 0.3%, 4.82%, and 4%, respectively, when using MRCI+P. In addition, our well depth shows an acceptable deviation of about 4% from the experimentally determined value by Yang et al. [51]. On the other hand, our data are in better agreement than those performed by Visscher et al. [20] at the CCSD(T)/cc-pVTZ level of theory. Our results show very slight deviations from high-level CCSD(T)/cc-pVQZ calculations [52], and from those described by Francesco et al. [53] at the SRCCSD(T) level of theory, which is widely regarded for its high accuracy. Liu et al. [18] underestimate the bond length $r_{e}$ compared to our value ($r_{e}=1.41\;Å$), which is closer to the experimental one. Our MRCI+Q value of $\omega_{e}$ is $906\;{cm}^{-1}$, which is closer to the experimental reference ($\omega_{e}=916.64\;{cm}^{-1}$) than any of the i_DMFT data [18]. The rotational constant value shows excellent agreement with the experimental data ($B_{e}=0.890\;{cm}^{-1}$), demonstrating consistency in both our results and the theoretical reference value.

For ${Cl}_{2}$, the well depths obtained from MRCI+Q and MRCI+P calculations, $D_{e}=20,282.36\;{cm}^{-1}$ and $D_{e}=20,249.10\;{cm}^{-1}$ respectively, display greater accuracy compared to the value reported by Peyrimhoff et al. ($D_{e}=20,076\;{cm}^{-1}$) [15]. These outcomes closely approximate the experimental binding energy of $D_{e}=20,242\;{cm}^{-1}$. Our results utilizing aug-cc-p5VZ at the MRCI level of theory demonstrate superior accuracy compared to the results obtained by Woon et al. [40], who utilized the same method but with a basis set lacking the diffuse layer cc-pV5Z. Notably, the values $r_{e}=1.99\;Å,\;D_{e}=20,249.10\;{cm}^{-1},\;\omega_{e}=548.34\;{cm}^{-1},\;\omega_{e}\chi_{e}=3.71\;{cm}^{-1},\;and\;B_{e}=0.238481\;{cm}^{-1}$ derived using MRCI+Q with aug-cc-pV5Z exhibit closer agreement with experimental data than those determined by Woon et al. at the same level of theory but with the cc-pVQZ basis set, as well as those found by Chattopadhyay et al. [17] at the SS-MRCC level of theory employing a cc-pVQZ basis set. This improvement in our findings is attributed to the larger basis set. Our dissociation energy with MRCI + *p* value ($20,282.36\;{cm}^{-1}$) differs by only about $6\;{cm}^{-1}$ from the best i_DMFT value reported by Liu et al. [18]. In addition, for all i_DMFT data, the vibrational frequency falls around $544.98\;{cm}^{-1}$ to $568.25\;{cm}^{-1}$, while our findings are $548.34\;{cm}^{-1}$ and $549.90\;{cm}^{-1}$, respectively, for MRCI+Q and MRCI+P, describing minimal deviations, especially when compared to experimental values, where the i_DMFT/cc-pVDZ value is the closest but still slightly underestimates the experimental value by about $14\;{cm}^{-1}$. For ${Cl}_{2}$, the MRCI+P results agree closely with the experimental data of Ref. [3], with deviations of 0.01 Å in $r_{e},\;5.56\;{cm}^{-1}$ in $D_{e},\;9.8\;{cm}^{-1}$ in $\omega_{e},\;1.045\;{cm}^{-1}$ in $\omega_{e}\chi_{e}$ and $0.00508\;{cm}^{-1}$ in $B_{e}$.

In the case of the ${Br}_{2}$ molecule, our computations of the target state incorporate higher electron correlation beyond the MRCI+Q level of theory and ECP28MWB, with 28 core electrons frozen for each atom, while the diatomic molecule is considered a system with 14 valence electrons, providing a reasonable depiction of bonds. The notable accord with experimental findings is evident [4,50]. Consequently, we identified an equilibrium distance of approximately 2.307 Å and a corresponding well depth of $16,204\;{cm}^{-1}$, indicating acceptable disparities. Compared with the experimental dissociation energy [50], our results using MRCI+Q and MRCI+P show differences of approximately 0.94% and 0.80%, respectively. When considering Focsa et al. [4] as a reference, our MRCI+Q and MRCI+P findings differ by approximately 0.92% and 0.78%, respectively. Although our $\omega_{e}$ values are lower by about 18 ${cm}^{-1}$, they compare well with the experimental results. In addition, our calculations yield equilibrium distances $r_{e}$, which agree well with literature values, with a maximum discrepancy of 0.1 Å. A similar conclusion applies to $B_{e}$, for which our value ($0.079\;{cm}^{-1})$ shows very good agreement with previously reported estimates.

In addition, our calculated ground-state properties of ${Br}_{2}$ agree closely with MRCISD+Q/aug-cc-pVTZ results, including SOC reported by Silva Gomes et al. [54], who obtained $r_{e}=2.279\;Å$ and $D_{e}=15,881.05\;{cm}^{-1}$ for the ${XO}_{g}^{+}$ state. Our values ($r_{e}=2.306\;Å$ and $D_{e}=16,204.79\;{cm}^{-1}$) deviate by only 1.19% and 2.30%, respectively. Furthermore, our results show excellent agreement with experimental data ($r_{e}=2.281\;Å$, and $D_{e}=16,054\;{cm}^{-1}$), with deviations of merely 1.10% for $r_{e}$ and 0.94% for $D_{e}$. When compared to SOC corrected theoretical values, the corresponding differences are 0.96% and 2.30%, respectively. These consistent small deviations indicate that spin-orbit coupling is negligible for the ${Br}_{2}$ ground state, confirming that the electronic structure is accurately described even without explicit inclusion of relativistic effects.

The reliability of the MRCI+Q calculations was further assessed through comparison with CCSD(T) results for the ground states of the homonuclear diatomic molecules. The same basis sets were used: cc-pV5Z, aug-cc-pV5Z, and ECP28MWB_AVTZ for $F_{2}$, ${Cl}_{2}$, and ${Br}_{2}$, respectively.

For $F_{2}$, both MRCI+Q and CCSD(T) reproduce the experimental equilibrium properties with good accuracy. MRCI+Q gives $r_{e}=1.413\;Å$, corresponding to a difference of 0.14%, compared to the experimental value $r_{e}=1.411\;Å$, while the CCSD(T) value $r_{e}=1.411\;Å$ shows no deviation from the experiment. Most notably, MRCI+Q predicts a dissociation energy of $12,821.68\;{cm}^{-1}$, which is much closer to the experimental value of $13,409.70\;{cm}^{-1}$ than the CCSD(T) result of $10,637.94\;{cm}^{-1}$. This represents deviations of 4.39% for MRCI+Q and 20.67% for CCSD(T), highlighting the better performance of MRCI+Q in describing the dissociation region of the $F_{2}$ potential energy while maintaining good accuracy near equilibrium.

For ${Cl}_{2}$, both methods provide comparable results, with MRCI+Q showing better agreement with the experiment for the dissociation energy. The MRCI+Q and CCSD(T) equilibrium bond lengths are 1.996 and 1.993 Å, respectively, compared with the experimental value of 1.980 Å. The MRCI+Q dissociation energy of 20,249.10 ${cm}^{-1}$ is in excellent agreement with the experimental value of 20,242 ${cm}^{-1}$, with a deviation of only 0.04%, whereas CCSD(T) gives 19,493.92 ${cm}^{-1}$, corresponding to a deviation of 3.70%.

For ${Br}_{2}$, the calculated bond lengths using MRCI+Q and CCSD(T) are 2.307 and 2.303 Å, respectively, with deviations of 1.14% and 0.96% from the experimental value of 2.281 Å, respectively. And for the dissociation energy, MRCI+Q predicts 16,204.76 ${cm}^{-1}$, with a deviation of only 0.92% from the experiment, whereas CCSD(T) gives 15,687.65 ${cm}^{-1}$, corresponding to a deviation of 2.30%. These results show that both methods provide a consistent description of ${Br}_{2}$, while MRCI+Q gives a more accurate dissociation energy.

Overall, the small deviations observed for equilibrium structures and spectroscopic constants confirm the reliability of the MRCI+Q approach. The differences in dissociation energies arise from the different correlation treatments employed by both methods.

#### 3.1.2. Heteronuclear Molecular Systems XO (X=F,Cl,Br)

**Figure 4:**
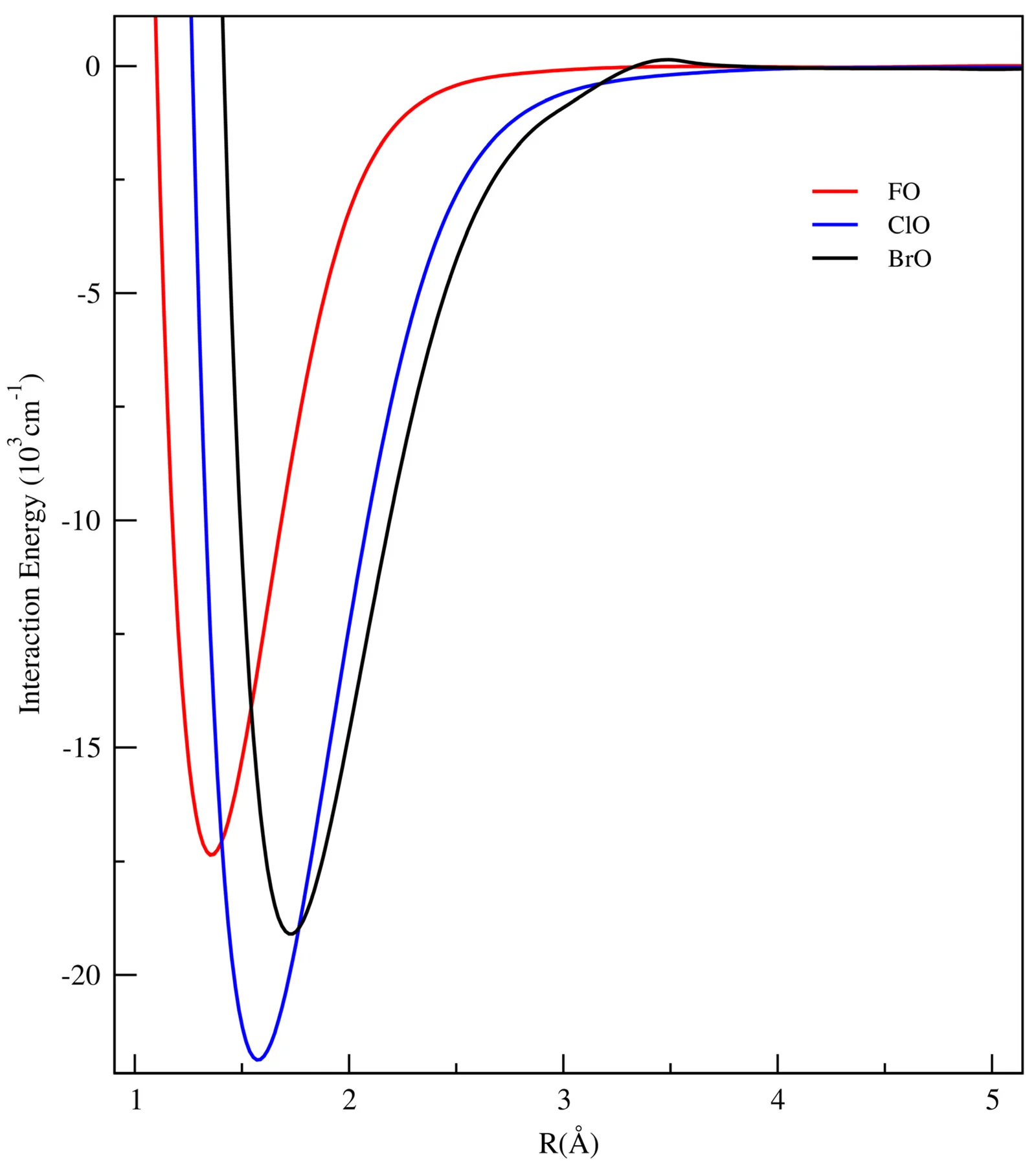
Potential energy curves of the heteronuclear diatomic radicals *FO*, *ClO*, and *BrO* in their ground electronic states ($1^{2}\Pi$), computed at the MRCI+Q level with the basis sets described in the text.

The same procedure was applied to the XO radicals, and the resulting PECs for *FO*, *ClO* and *BrO* are shown in Figure 4. Compared with the homonuclear $X_{2}$ molecules, the XO radicals generally exhibit shorter equilibrium bond lengths and larger dissociation energies.

The spectroscopic parameters (the equilibrium bond length $r_{e}$, dissociation energy $D_{e}$, harmonic frequency $\omega_{e}$, anharmonicity constant $\omega_{e}\chi_{e}$, and rotational constant $B_{e}$) are gathered in Table 2 and compared with existing theoretical and experimental works. The enhanced bond strengths and shorter bond lengths observed in XO molecules compared to their $X_{2}$ counterparts arise from the substantial electronegativity difference between X and O. This difference imparts significant ionic character to the heteronuclear X−O bond, introducing electrostatic attraction that reinforces the covalent interaction. Within the XO series, the bonding behavior follows the same general pattern observed in the $X_{2}$ series. Although atomic size largely controls bond length, giving BrO the longest bond and FO the shortest, the strongest bond occurs in ClO. Chlorine achieves the best balance: its size allows effective orbital overlap while keeping lone-pair repulsion moderate. In contrast, although the FO bond is stronger than that in $F_{2}$, it is limited by significant lone-pair repulsion between the small fluorine and oxygen atoms, whereas the BrO bond, though more stable than ${Br}_{2}$, remains weaker than that in ClO because bromine’s large radius reduces the efficiency of orbital overlap with oxygen.

The spectroscopic parameters obtained at the MRCI+Q level are in good agreement with the available experimental and theoretical data.

Specifically, for the FO dimer, the calculated equilibrium distance $r_{e}=1.357\;Å$ and the rotational constant $B_{e}=1.054054\;{cm}^{-1}$ are in excellent agreement with all available references. Our calculations with MRCI+Q in conjunction with the cc−pVQZ basis set yielded a well depth equal to $17,360.68\;{cm}^{-1}$, underestimated by several hundreds of ${cm}^{-1}$ compared to those found by Li et al. [56] at the same level of theory with the AV5Z and AV6Z basis sets, and differ from the experimental value by 2.47% [23]. Accordingly, the observed differences may arise from the smaller basis set used in our simulation, which may not fully capture the electronic correlation effects. The harmonic frequency predicted by our computations closely matches the experimental value of $1053\;{cm}^{-1}$ [23], outperforming other available theoretical values and indicating the accuracy of our approaches.

For the ClO molecular system, the spectroscopic constants $r_{e}=1.575\;Å$ and $\omega_{e}=842\;{cm}^{-1}$ calculated using the MRCI+Q and MRCI+P levels of theory with the aug-cc-pV6Z basis set show good agreement with the data previously reported by Wang et al. [24]. Moreover, the well depth $D_{e}=21,872.61\;{cm}^{-1}$ obtained from these calculations is slightly underestimated compared to that found by Li et al. [26]. Furthermore, the dissociation energy obtained using the MRCI+Q method with the aug-cc-pV6Z basis set yields the highest accuracy, indicating the reliability of our calculations. The relative differences compared to the experimental results [50,59] are 1.4% and 2.57%, respectively. In addition, calculations using the MRCI+P method yielded spectroscopic parameters very similar to those obtained with MRCI+Q calculations, as shown in Table 2, when employing the aug-cc-pV6Z basis set. This indicates notable precision, with differences of approximately 1.87% and 3.04% in the dissociation energy $D_{e}$ relative to experimental data [50,59].

For *BrO*, the binding energies were computed at the MRCI+Q and MRCI+P levels using ECP28MWB/aug-cc-pVQZ for Br and cc-pVQZ for *O*. The pseudopotential reduces the number of explicitly treated electrons and makes the calculations computationally tractable. Our strong binding energies of $D_{e}=19,107.13\;{cm}^{-1}$ and $19,011.32\;{cm}^{-1}$ describe excellent agreement compared to the experimental value [55], with differences of about 0.48% and 0.98% for MRCI+Q and MRCI+P, respectively. Nevertheless, the equilibrium distances $r_{e}$ exhibit minimal variances compared to those determined by Balabanov et al. [61] at the high-level CCSD(T) calculation. Furthermore, our computed harmonic frequency $\omega_{e}=708\;{cm}^{-1}$ is underestimated by around $20\;{cm}^{-1}$ compared with both experimental [60] and theoretical [61] values.

### 3.2. Vibrational Levels of Homonuclear $X_{2}$ and Heteronuclear XO Complexes

The discrete-variable representation (DVR) method was used to calculate vibrational energy levels and level spacings for the $X_{2}$ and XO diatomic species. This numerical technique is well-suited for solving the one-dimensional nuclear Schrödinger equation using a set of discrete grid points along the internuclear coordinate. For each molecule, the DVR calculation used the corresponding ab initio PEC as the input potential. In the case of homonuclear molecules ($X_{2}$ with *X* = *F*, *Cl*, *Br*), the DVR method was applied along the symmetric X−X bond coordinate. For the heteronuclear species (XO), the DVR was applied along the X−O bond.

All the vibrational energies E(*v*) and the corresponding energy spacings E(*v*) − E(*v* − 1) of the ground electronic states 1$\sum_{g}^{+}$ for $X_{2}$ and $1^{2}\Pi$ for XO are provided in the Supplementary Materials (see Table S1 and Table S2, respectively). The behavior of these vibrational spacings reflects the anharmonicity of the potential energy wells. Unlike the harmonic oscillator model, where levels are equally spaced, the vibrational levels in these real molecular systems are non-equidistant and become increasingly closer as the vibrational quantum number *v* increases. As a result, the energy spacing ΔE gradually decreases with increasing *v*, especially near the dissociation limit, where the potential flattens. In our calculations, we identified 22, 56, and 82 vibrational levels for $F_{2},\;{Cl}_{2}$ and ${Br}_{2}$ respectively. For the heteronuclear species, the number of vibrational levels found is 27 for FO, 44 for ClO, and 42 for BrO.

Differences in the number of bound vibrational levels arise from variations in well depth and reduced mass: deeper wells and larger reduced masses generally support more bound states.

### 3.3. PESs of $X_{2}O$ (X = F, Cl, and Br) Triatomic Molecules

As mentioned in Section 2 the potential energy surfaces (PESs) for the triatomic complexes were calculated using two distinct Jacobi coordinate configurations shown in Figure 1. First, the PESs were calculated for several fixed internuclear distances of the $X_{2}$ dimer (Figure 1a): from 1.6 to 2.8 Å for $F_{2}$, from 2.2 to 3.4 Å for ${Cl}_{2}$, and from 2.4 to 3.6 Å for ${Br}_{2}$, using a uniform step size of 0.2 Å. This approach is based on the observation that the electronic cloud of the $X_{2}$ dimer is only perturbed by the presence of the O atom. On the other hand, the X−O bond was fixed while varying the distance between the remaining X atom and the center of mass of the XO fragment (Figure 1b). To capture the effects of bond flexibility, the X−O bond lengths were sampled over the ranges 1.0–1.8 Å for FO, 1.3–2.1 Å for ClO, and 1.4–2.2 Å for BrO, again using a step of 0.2 Å. It is worth noting that the bond distance grid associated with the diatomic fragment of the triatomic molecule was defined around the equilibrium bond, taken from available experimental data, ensuring a reliable description of the molecular geometry in the vicinity of equilibrium.

For each fixed geometry, the ground singlet electronic state energy of our systems was calculated using the MRCI+Q level of theory to account for electron correlation effects accurately. All calculations were carried out with the correlation-consistent AVQZ. The resulting PESs were thus obtained for the different values of r. We observe that for each angular orientation θ, even a small change in r significantly influences the interaction energy of the total complex.

In the first investigation, for all $F_{2}O$, ${Cl}_{2}O$, and ${Br}_{2}O,$ the T-shaped geometry (θ = 90°) is more stable than the linear geometries. In Tables S3–S5 (Supplementary Materials) we have summarized all spectroscopic constants: the distances R (Å) and the minimum energies $E_{min}$ (${cm}^{-1}$), harmonic frequency $\omega_{e}$ (${cm}^{-1}$), and the equilibrium rotational constant $B_{e}$ (${cm}^{-1}$) for the $X_{2}O$ complex configurations for all different orientations, across different values of r calculated using the MRCI+Q approach with AVQZ basis. The equilibrium geometries reported in these tables were obtained by cubic-spline interpolation of the ab initio grid points along R, followed by BFGS optimization. Basin-hopping global optimization was used to verify the stability of each minimum. From Table 3, a comparative analysis of the most stable configuration, where θ = 90° for the $F_{2}O$, ${Cl}_{2}O$, and ${Br}_{2}O$ complexes, reveals a consistent trend in how the bond distance R and minimum energies $E_{min}$ (${cm}^{-1}$) vary with the internuclear distance r of the $X_{2}$ molecule. For all three systems, increasing r leads to a decrease in R, indicating a more compact geometry, and to an initial decrease in energy $E_{min}$, reflecting modest $O-X_{2}$ interactions.

As mentioned before, the potential energy surface analysis for all $X_{2}O$ systems (X=F,Cl, and Br) consistently identifies the perpendicular configuration, θ = 90°, as the global minimum. The bent geometry of $X_{2}O$ gives rise to a permanent dipole moment arising from the electronegativity difference between oxygen and the halogen atoms. The electronic structure is dominated by σ bonding interactions between the oxygen p orbitals and the halogen p orbitals, with additional π-type contributions that enhance bond stabilization. Within a molecular orbital framework, the highest occupied molecular orbital (HOMO) is more localized and characterized by non-bonding contributions from oxygen. In contrast, the lowest unoccupied molecular orbital (LUMO) exhibits antibonding character along the O−X bonds, governing the low-lying electronic excitations and chemical reactivity. $F_{2}O$ exhibits, at $r_{e}=2.20\;Å$ and $R_{e}=0.85\;Å$, a minimum interaction energy equal to −41,936.89 ${cm}^{-1}$ (Table 3). These findings align well with experimental bond length values $r_{e}=2.20\;Å$ and $R_{e}=0.88\;Å$ [62] (Table 4). In addition, for ${Cl}_{2}O$, the potential minimum is located at $r_{e}=2.80\;Å$ and $R_{e}=0.95\;Å$, corresponding to an interaction energy of approximately $E_{min}=-43,303.62\;{cm}^{-1}$ (Table 3). The minimum energy grid point is in very good agreement with experimental observations, which report a Cl−Cl bond length of 2.80 Å and a Cl−O distance of 0.96 Å, close to the predicted values [63] (Table 4). Similarly, for ${Br}_{2}O$, the minimum identified within the sampled ab initio grid is situated at $r_{e}=3.00\;Å$ and $R_{e}=1.029\;Å$, with a minimum energy of about $E_{min}=-42,048.66\;{cm}^{-1}$ (Table 3), consistent with the experimental Br−Br bond length $r_{e}=3.06\;Å$ and Br−O bond length $R_{e}=1.03\;Å$ [64] (Table 4).

In the second investigation, across the XO−X series, the analysis reveals a significant change in preferred angular stability. The results of our XO−X configurations are listed in Tables S6–S8 (Supplementary Materials). The potential energy minima occur in our grid at θ=50.0327° for FO−F and at θ=36.7189° for ClO−Cl and BrO−Br, each corresponding to their respective optimized bent geometries. Table 5 collects the spectroscopic parameters of the most stable configurations for the FO−F, ClO−Cl, and BrO−Br complexes. For FO−F, it displays a potential well with a minimum at $r_{e}=1.4\;Å$ and $R_{e}=1.738\;Å$ and an interaction energy of $-41,899.88\;{cm}^{-1}$ (Table 5). This equilibrium geometry of θ=50.0327° agrees closely with experimental findings that report θ=51.54° and an F−O bond length of 1.41 Å and a distance of 1.75 Å between F and the center of mass of FO [62] (Table 6). Moreover, for ClO−Cl, θ=36.7189° represents the most stable angular orientation on the chosen grid, which is considered to be close to the experimental value, θ=42.02°. At this optimal geometry, the minimum is located at $r_{e}=1.7\;Å$ and $R_{e}=2.5\;Å,$ with an interaction energy of about $E_{min}=-42,777.41{cm}^{-1}$ (Table 5), which is in good agreement with experimental data that report a Cl-O bond length of 1.69 Å and a distance of 2.36 Å between Cl and the center of mass of the Cl-O bond [63] (Table 6). Finally, BrO−Br also exhibits its minimum at θ=36.7189° on our grid, and $r_{e}=1.8\;Å$ and $R_{e}=2.8\;Å,$ with a minimum energy of −42,054.06 ${cm}^{-1}$ (Table 5). The calculated geometry aligns well with experimental measurements, which give θ=37.58°, a Br−O bond length of 1.76 Å, and a distance of 2.67 Å between Br and the center of mass of BrO [64] (Table 6), respectively.

Across all systems studied, the $X_{2}O$ and XO−X representations yield comparable minima, indicating that the two coordinate descriptions are mutually consistent. Using the highest energy as a reference, in the lighter $F_{2}O$ and FO−F pair, the difference between configurations is small, around 0.09%. Even for ${Cl}_{2}O$ and ClO−Cl, the relative deviation between the two energies is on the order of 1.22%, while for ${Br}_{2}O$ and BrO−Br, the difference narrows even further, to about 0.012%, indicating an almost identical energetic landscape. These small energy differences support the internal consistency of the calculated PESs and suggest that the present surfaces should be suitable for future dynamical and reactivity studies.

**Figure 5:**
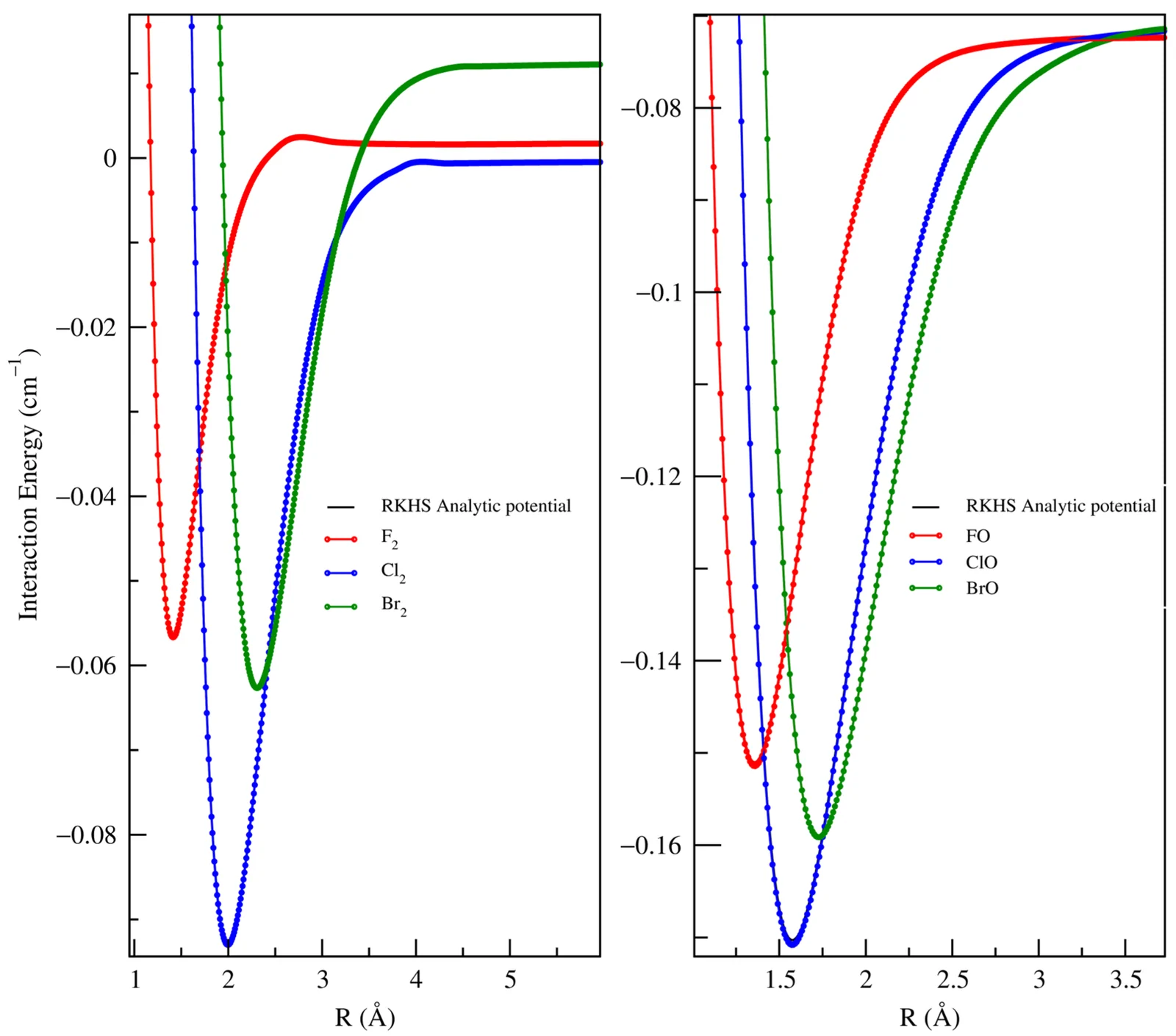
Comparison between the analytical potential energy curves obtained via RKHS interpolation and the ab initio data for $X_{2}$ and XO molecules.

Figure 5 compares the RKHS-interpolated potentials with the underlying ab initio data for representative $X_{2}O$ and XO−X cuts. The results demonstrate that the RKHS potential accurately represents the potential energy surface (PES) across short-, intermediate-, and long-range regions. The performance of each kernel was evaluated using the root mean square error (RMSE), which quantifies the deviation between the RKHS-interpolated energies and the original ab initio MRCI+Q/AVQZ values. Our root mean square (RMSE) error is on the order of 10^−6^ Hartree, and it is computed as follows: $\sqrt{\frac{\sum_{i=1}^{n}{\left(y_{i}-{\hat{y}}_{i}\right)}^{2}}{n}}$, where *n* represents the number of ab initio points, and $y_{i}$ and ${\hat{y}}_{i}$ denote the actual interpolated values and the predicted ab initio values, respectively. Among the tested kernels, the selected form produced the lowest RMSE while preserving physically reasonable long-range behavior and was therefore adopted for all final PES representations.

**Figure 6:**
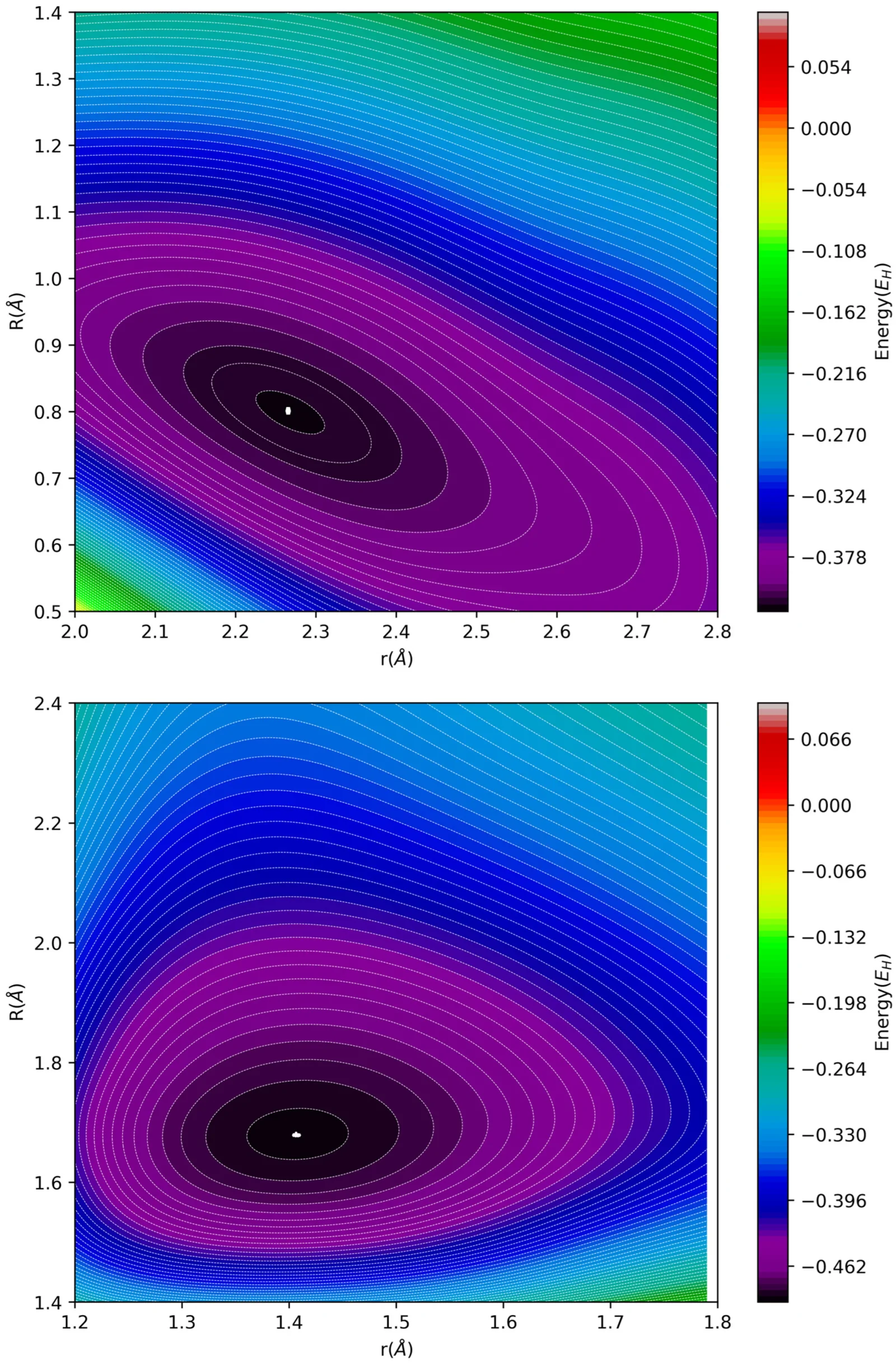
Two-dimensional contour plot of the analytical potential using the RKHS of the total energy minimum of MRCI+Q ab initio data in the (r, R) plane for $F_{2}O$ and FO−F configurations.

**Figure 7:**
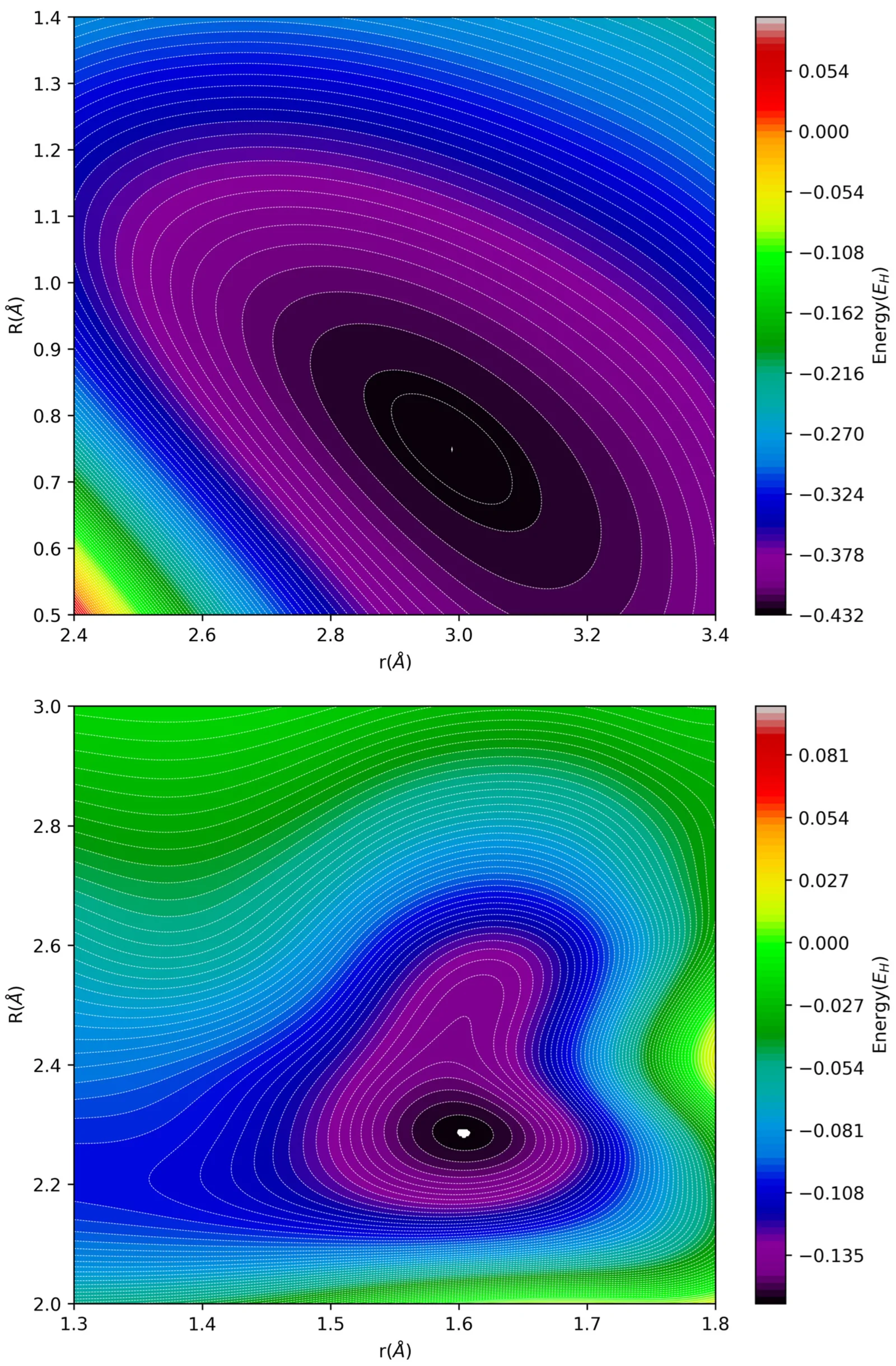
Two-dimensional contour plot of the analytical potential using the RKHS of the total energy minimum of MRCI+Q ab initio data in the (r, R) plane for ${Cl}_{2}O$ and ClO−Cl configurations.

**Figure 8:**
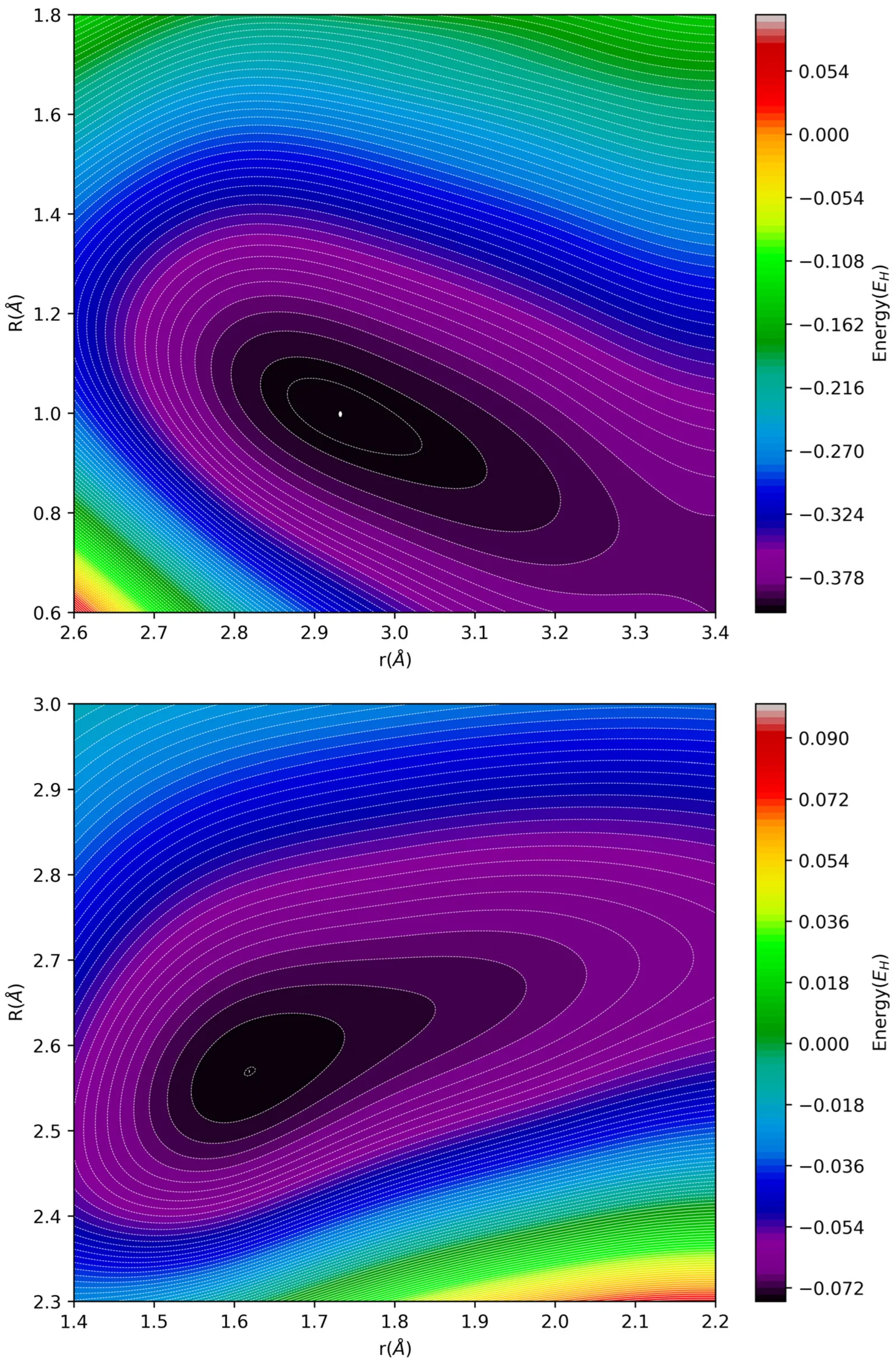
Two-dimensional contour plot of the analytical potential using the RKHS of the total energy minimum of MRCI+Q ab initio data in the (r, R) plane for ${Br}_{2}O$ and BrO−Br configurations.

Figure 6, Figure 7 and Figure 8 show two-dimensional contour plots of the RKHS potential in the relevant coordinate planes for both the $X_{2}O$ and XO−X configurations. The color scale represents energy in Hartree units ($E_{h}$), with lower energy in darker shades (purple/blue) and higher energies in green/yellow. A white dot at the coordinates indicates the absolute minimum on the PES. This point corresponds to the minimum on the PES and therefore to the most stable geometry of the complex. For the $X_{2}O$ configuration, the molecules achieve their lowest potential energy configurations at $r_{e}=2.27\;Å$ and $R_{e}=0.80\;Å$ for $F_{2}O$, $r_{e}=2.99\;Å$ and $R_{e}=0.75\;Å$ for ${Cl}_{2}O$, and $r_{e}=2.93\;Å$ and $R_{e}=1.00\;Å$ for ${Br}_{2}O$ when using the MRCI+Q level of theory (see Table 4). Furthermore, it is worth mentioning that RKHS results closely align with previously reported experimental values, where the equilibrium X−X bond lengths $r_{e}$ and X−O bond lengths R fall within ($r_{e}=$ 2.20 Å and $R_{e}$ = 0.88 Å) [62], ($r_{e}=$ 2.80 Å and $R_{e}$ = 0.96 Å) [63], and ($r_{e}=$ 3.06 Å and $R_{e}=1.03\;Å)$ [64] for $F_{2}O$, ${Cl}_{2}O$, and ${Br}_{2}O$, respectively. Table 4 summarizes the RKHS results and compares them with available experimental and ab initio data for bond lengths. Furthermore, as mapped in Figure 6, Figure 7 and Figure 8, the contour plots of our molecules in the XO−X configuration possess prominent minima at $r_{e}=1.41\;Å$ and $R_{e}=1.68\;Å$ for FO−F, at $r_{e}=1.63\;Å$ and $R_{e}=2.26\;Å$ for ClO−Cl, and at $r_{e}=1.62\;Å$ and $R_{e}=2.57\;Å$ for BrO−Br, marked by a deep, dark purple attractive well. These structural features, as shown in Table 6, are consistent with the experimental and ab initio minimum geometries found at $r_{e}=1.41\;Å$ and $R_{e}=1.75\;Å$ [62], at $r_{e}=1.69\;Å$ and $R_{e}=2.36\;Å$ [63], and at $r_{e}=1.76\;Å$ and $R_{e}=2.67\;Å$ [64] for FO−F, ClO−Cl, and BrO−Br, respectively.

In summary, the locations of the minima are consistent with the expected structural trends, supporting the reliability of the present PESs and the underlying ab initio calculations.

## 4. Conclusions

In this work, we have performed a systematic high-level ab initio investigation of the ground-state electronic structure and spectroscopic properties of the diatomic molecules $X_{2}$ and XO, and the triatomic complexes $X_{2}O$, where X=F, Cl, and Br. Using multireference configuration interaction (MRCI+Q and MRCI+P) methods with large correlation-consistent basis sets, we obtained spectroscopic constants that are generally in good agreement with available reference data.

For the diatomic species, the computed spectroscopic constants, including equilibrium bond lengths, dissociation energies, harmonic frequencies, and anharmonicity constants, show good agreement with available experimental data. Relative deviations in dissociation energies are remarkably low: 4.00% for $F_{2}$, 0.03% for ${Cl}_{2}$, 0.92% for ${Br}_{2}$, 2.46% for FO, 1.40% for ClO, and 0.48% for BrO. These results establish a reliable benchmark for halogen-containing diatomics and provide a solid foundation for modeling the corresponding triatomic systems.

For the triatomic $X_{2}O$ complexes, we constructed accurate potential energy surfaces (PESs) using two complementary Jacobi coordinate representations. The MRCI+Q/aug-cc-pVQZ data were interpolated using the reproducing kernel Hilbert space (RKHS) method, yielding smooth analytical surfaces with root mean square errors on the order of 10^−6^ Hartree. Our calculations accurately reproduce the experimental equilibrium geometries of $F_{2}O,\;{Cl}_{2}O$, and $Br_{2}O,$ with bond lengths deviating by less than 0.3 Å from the reported values. The two configurational representations ($X_{2}O$ and XO−X) yield remarkably consistent interaction energies, with relative deviations below 1.2% across all systems, confirming the internal consistency and robustness of our PESs.

The high-quality PESs and spectroscopic constants reported here provide a valuable resource for future studies, including molecular dynamics simulations, reaction dynamics, and investigations of halogen oxide chemistry in atmospheric and environmental contexts. Ongoing work focuses on extending this methodology to excited electronic states and incorporating spin–orbit coupling effects, which are essential for a complete understanding of the electronic structure and photochemical behavior of these environmentally significant molecules.

## Figures and Tables

**Table 1:** Spectroscopic parameters of the ground ($1\sum_{g}^{+}$) electronic states of $F_{2}$, ${Cl}_{2}$ and ${Br}_{2}$ diatomic molecules.

			$F_{2}$				
**Method**	**Basis Set**	$r_{e}(Å)$	$D_{e}({\mathrm{cm}}^{-1})$	$\omega_{e}({\mathrm{cm}}^{-1})$	$\omega_{e}\chi_{e}({\mathrm{cm}}^{-1})$	$B_{e}({\mathrm{cm}}^{-1})$	**Reference**
MRCI	*cc*-pV5Z	1.414	12,040.98	888.19	16.37	0.887586	This work
MRCI+Q		1.413	12,821.68	906.25	16.01	0.888843	This work
MRCI+P		1.414	12,763.36	902.69	15.96	0.887586	This work
CCSD(T)	cc-pV5Z	1.411	10,637.94	919.22	62.93	0.891365	This work
		1.411	13,409.70	916.64	11.23	0.890000	Exp [50]
			13,409.60				Exp [51]
CAS-SCF		1.412	13,493.60	948.00			[12]
SS-MRCCSD	cc-pVQZ	1.410	14,270.00	925.40	11.70		[17]
i_DMFT	cc-pVDZ	1.409	13,410.60	894.91	10.67	0.894000	[18]
	cc-pVTZ	1.384	13,412.10	996.78	13.27	0.927000	
	cc-pVQZ	1.378	13,412.90	989.89	12.80	0.935000	
	Aug-cc-pV5Z	1.347	13,409.60	1058.32	14.22	0.978000	
CCSD(T)	cc-pVTZ	1.416	12,171.00	919.00			[20]
CCSD(T)	cc-pVQZ	1.413	12,836.00	921.00			[52]
SR-CCSD(T)	cc-pV5Z	1.408	13,186.00	935.00	10.80		[53]
			${Cl}_{2}$				
Method	Basis **Set**	$r_{e}(Å)$	$D_{e}({\mathrm{cm}}^{-1})$	$\omega_{e}({\mathrm{cm}}^{-1})$	$\omega_{e}\chi_{e}({\mathrm{cm}}^{-1})$	$B_{e}({\mathrm{cm}}^{-1})$	Reference
MRCI	Aug-cc-pV5Z	1.997	19,602.36	546.73	3.81	0.238481	This work
MRCI+Q		1.996	20,249.10	548.34	3.71	0.238481	This work
MRCI+P		1.996	20,282.36	549.90	3.72	0.238720	This work
CCSD(T)	Aug-cc-pV5Z	1.993	19,493.92	556.01	5.38	0.239439	This work
		1.987	20,276.80	559.70	2.68	0.243800	Exp [3]
		1.980	20,242.00	564.90	4.00	0.243800	Exp [50]
MRD-CI		1.988	20,076.00	552.00			[15]
SS-MRCCSD	cc-pVQZ	1.993	19,754.20	556.18	2.12		[17]
i_DMFT	cc-pVDZ	2.031	20,275.70	544.98	2.93	0.234000	[18]
	cc-pVTZ	2.012	20,281.80	554.70	2.59	0.238000	
	cc-pVQZ	2.002	20,280.60	557.14	2.63	0.241000	
	Aug-cc-pV5Z	1.992	20,279.20	568.25	2.75	0.243000	
MRCI	cc-pV5Z	1.996	19,421.90	554.60	2.81	0.242000	[40]
MRCI+Q	cc-pVQZ	2.005	19,467.40	549.90	2.76	0.240000	
			${Br}_{2}$				
Method	Basis **Set**	$r_{e}(Å)$	$D_{e}({\mathrm{cm}}^{-1})$	$\omega_{e}({\mathrm{cm}}^{-1})$	$\omega_{e}\chi_{e}({\mathrm{cm}}^{-1})$	$B_{e}({\mathrm{cm}}^{-1})$	Reference
MRCI	ECP28MWB_AVTZ	2.306	15,796.29	307.27	1.49	0.079349	This work
MRCI+Q		2.307	16,204.76	307.79	1.46	0.079280	This work
MRCI+P		2.307	16,183.06	307.84	1.46	0.079280	This work
CCSD(T)	ECP28MWB_AVTZ	2.303	15,687.65	311.04	2.13	0.079555	This work
		2.281	16,056.90	325.32	1.08	0.082000	Exp [4]
		2.281	16,054.00	325.30			Exp [50]
SS-MRCCSD	cc-pVQZ	2.280	19,327.50	327.38	1.50		[17]
i_DMFT	cc-pVDZ	2.332	16,060.40	308.49	1.09	0.079000	[18]
	cc-pVTZ	2.313	16,059.50	316.14	1.03	0.080000	
	cc-pVQZ	2.305	16,056.00	317.57	1.05	0.080000	
	Aug-cc-pV5Z	2.294	16,068.70	322.51	1.07	0.081000	
	pVTZ	2.315	16,124.00	316.00			[20]
CCSD(T)	AV5Z	2.296		326.10			[54]
CCSD(T)	AVQZ	2.296		323.70			

**Table 2:** Spectroscopic parameters of the $1^{2}\Pi$ ground state of FO, ClO, and BrO diatomic molecules.

			FO				
Method	Basis Set	$r_{e}(Å)$	$D_{e}({\mathrm{cm}}^{-1})$	$\omega_{e}({\mathrm{cm}}^{-1})$	$\omega_{e}\chi_{e}({\mathrm{cm}}^{-1})$	$B_{e}({\mathrm{cm}}^{-1})$	Reference
MRCI		1.355	16,556.98	1057.42	16.88	1.057167	**This work**
MRCI+Q	cc-pVQZ	1.357	17,360.68	1060.12	16.18	1.054054	**This work**
MRCI+P		1.357	17,273.58	1059.20	16.23	1.054054	**This work**
		1.354		1053.00	9.91	1.058707	**Exp** [23]
			17,800.00				**Exp** [55]
MRCI+Q	AVQZ		17,698.00				[27]
MRCI+Q	AV5Z	1.354	17,857.00	1090.25			[56]
MRCI+Q	AV6Z	1.355	17,945.80	1091.73			
QCISD		1.356		1026.70	22.30	1.056000	[57]
MRCI		1.355		1047.10	10.40	1.056700	[58]
			ClO				
Method	Basis Set	$r_{e}(Å)$	$D_{e}({\mathrm{cm}}^{-1}$)	$\omega_{e}({\mathrm{cm}}^{-1})$	$\omega_{e}\chi_{e}({\mathrm{cm}}^{-1})$	$B_{e}({\mathrm{cm}}^{-1})$	Reference
MRCI	Aug-cc-pV6Z	1.578	20,694.91	830.31	8.32	0.614116	This work
MRCI+Q		1.575	21,872.61	842.42	8.11	0.616458	This work
MRCI+P		1.575	21,767.90	842.35	8.14	0.616458	This work
		1.569	22,450.38	853.80	5.50	0.623440	Exp [50]
		1.569	22,183.46	853.80	5.80	0.623340	Exp [59]
icMRCI+Q	aug-cc-pV5Z	1.577	22,515.64	842.30			[24]
MRCI+Q/Q5/+CV+DK		1.564	22,088.97	845.82	9.12	0.627750	
MRCI	AV6Z	1.569	21,470.50	852.54	5.77	0.623760	[26]
			BrO				
**Method**	**Basis Set**	$r_{e}(Å)$	$D_{e}({\mathrm{cm}}^{-1})$	$\omega_{e}({\mathrm{cm}}^{-1})$	$\omega_{e}\chi_{e}({\mathrm{cm}}^{-1})$	$B_{e}({\mathrm{cm}}^{-1})$	**Reference**
MRCI	ECP28MWB_AVQZ	1.731	18,032.99	699.53	6.78	0.422059	**This work**
MRCI+Q	cc-pVQZ	1.728	19,107.13	708.29	6.56	0.423526	**This work**
MRCI+P		1.728	19,011.32	708.19	6.59	0.423526	**This work**
			19,200.00				**Exp** [55]
		1.717		725.42			**Exp** [60]
MRCI+Q	AVQZ		19,341.00				[27]
CCSD(T)	AVQZ	1.725		726.00			[54]
CCSD(T)	AV5Z	1.722		729.60			

**Table 3:** Spectroscopic parameters of different intermolecular distances of $F_{2}O$, ${Cl}_{2}O$, and ${Br}_{2}O$, at θ = 90° using the AVQZ basis set and MRCI+Q level of theory.

r(Å)	R(Å)	$E_{min}({cm}^{-1})$	$\omega_{e}{(cm}^{-1})$
	$F_{2}O$		
1.6	2.576	−16,175.04	76.48
1.8	0.986	−33,660.80	1079.71
2.0	0.919	−40,569.10	1012.37
2.2	0.853	−41,936.89	931.36
2.4	0.788	−40,095.01	848.57
2.6	0.726	−36,269.96	763.29
2.8	0.667	−31,222.34	678.54
	${Cl}_{2}O$		
2.2	1.204	−28,440.61	783.73
2.4	1.120	−37,759.67	750.18
2.6	1.034	−42,182.30	688.73
2.8	0.948	−43,303.62	623.23
3.0	0.866	−42,086.36	563.10
3.2	0.794	−39,146.32	513.93
3.4	0.735	−34,978.74	474.13
	${Br}_{2}O$		
2.4	1.309	−27,408.97	694.69
2.6	1.219	−36,036.37	655.58
2.8	1.126	−36,081.36	604.64
3.0	1.029	−42,048.66	547.83
3.2	0.930	−41,604.44	491.12
3.4	0.842	−39,545.55	440.25
3.6	0.766	−36,196.58	398.94

**Table 4:** Intermolecular distances within $X_{2}$ ($r_{e}$) between O and the center of mass of $X_{2}$ ($R_{e}$) of the $F_{2}O$, ${Cl}_{2}O$, and ${Br}_{2}O$ complexes obtained from the MRCI+Q/AVQZ calculations at the optimal angular orientation θ = 90°.

Molecule	$r_{e}\;(Å)$	$R_{e}\;(Å)$	References
$F_{2}O$	2.20	0.88	[62]
	2.20	0.85	Ab initio
	2.27	0.80	RKHS
${Cl}_{2}O$	2.80	0.96	[63]
	2.80	0.95	Ab initio
	2.99	0.75	RKHS
${Br}_{2}O$	3.06	1.03	[64]
	3.00	1.03	Ab initio
	2.93	1.00	RKHS

**Table 5:** Spectroscopic parameters of different intermolecular distances r of FO−F, ClO−Cl, and BrO−Br using the AVQZ basis set and MRCI+Q level of theory.

r(Å)	R (Å)	$E_{min}({cm}^{-1})$	$\omega_{e}{(cm}^{-1})$
	***FO-F***		
1.0	1.790	4895.26	644.29
1.2	1.742	−35,272.51	735.19
1.4	1.738	−41,899.88	790.36
1.6	1.746	−38,239.33	795.35
1.8	1.757	−31,923.55	773.43
	***ClO-Cl***		
1.3	2.469	−14,363.71	257.80
1.5	2.400	−38,326.04	473.05
1.7	2.448	−42,777.41	519.04
1.9	2.497	−40,356.89	516.64
2.1	2.538	−36,093.38	509.71
	***BrO-Br***		
1.4	2.767	−8561.79	183.76
1.6	2.734	−35,887.03	305.53
1.8	2.792	−42,054.06	323.96
2.0	2.853	−40,319.65	323.22
2.2	2.905	−36,107.64	309.59

**Table 6:** Interatomic distances within XO ($r_{e}$) and intermolecular distances between X and the center of mass of XO ($R_{e}$) for the FO−F, ClO−Cl, and BrO−Br complexes obtained from the MRCI+Q/AVQZ calculations at their optimal angular orientations.

Molecule	$r_{e}(Å)$	$R_{e}(Å)$	References
FO−F	1.41	1.75	[62]
	1.40	1.74	Ab initio
	1.41	1.68	RKHS
ClO−Cl	1.69	2.36	[63]
	1.70	2.45	Ab initio
	1.63	2.26	RKHS
BrO−Br	1.76	2.67	[64]
	1.80	2.79	Ab initio
	1.62	2.57	RKHS

## Data Availability

The original contributions presented in this study are included in the article/Supplementary Material. Further inquiries can be directed to the corresponding author(s).

## Supplementary Materials

The following supporting information can be downloaded at https://www.mdpi.com/article/10.3390/molecules31173035/s1: Table S1: Vibrational energy levels trapped in the ground state ^1^$\sum_{g}^{+}$ of our homonuclear systems, using MRCI+Q level of theory with basis sets cc-pV5Z, aug-cc-pV5Z, and ECP28MWB_AVTZ for $F_{2}$, ${Cl}_{2}$, and ${Br}_{2}$, respectively; Table S2: Vibrational energy levels trapped in the ground state ^2^Π of our heteronuclear systems, using MRCI+Q level of theory with basis sets cc-pVQZ, aug-cc-pV6Z, and ECP28MWB_AVQZ/cc-pVQZ for FO, ClO, and BrO, respectively; Table S3: Spectroscopic constants of the ground state of $F_{2}O$ using MRCI+Q/AVQZ for different Jacobi angles θ and bond lengths *r* and *R*; Table S4: Spectroscopic constants of ${Cl}_{2}O$ configuration using MRCI+Q/AVQZ for different Jacobi angles θ and bond lengths *r* and *R*; Table S5: Spectroscopic constants of ${Br}_{2}O$ using MRCI+Q/AVQZ for different Jacobi angles θ and bond lengths *r* and *R*; Table S6: Spectroscopic constants of FO−F using MRCI+Q/AVQZ for different Jacobi angles θ and bond lengths *r* and *R*; Table S7: Spectroscopic constants of ClO−Cl using MRCI+Q/AVQZ for different Jacobi angles θ and bond lengths *r* and *R*; Table S8: Spectroscopic constants of BrO−Br using MRCI+Q/AVQZ for different Jacobi angles θ and bond lengths *r* and *R*.
