# Supplementary material for: Benchmark MRCI Study of Halogen Oxides: Potential Energy Surfaces and Spectroscopic Constants for X2, XO, and X2O (X = F, Cl, Br)
Source: Molecules. 2026 Aug 28;31(17):3035. doi: 10.3390/molecules31173035 (PMC13567641; doi:10.3390/molecules31173035)
Supplement: Supplementary file 1 [file molecules-31-03035-s001.zip › molecules-4471226-supplementary.pdf]

Supplementary Materials

Benchmark MRCI Study of Halogen Oxides: Potential Energy Surfaces and Spectroscopic Constants for X_2, XO, and X_2O (X = Cl, Br, F)

Imen Selmi[1], Mohamed Bejaoui[1,2], Jamila Dhiflaoui[1, 2], Patryk Jasik[3,4,5], Józef E Sienkiewicz[3], and Hamid Berriche [1,3,6, *]

[1]University of Monastir, Faculty of Science of Monastir, Laboratory of Interfaces and Advanced Materials LR11ES55, Physics Department, 5019 Monastir, Tunisia

[2]University of Monastir, Preparatory Institute for Engineering Studies of Monastir, Physics Department, 5019 Monastir, Tunisia.

[3] Faculty of Applied Physics and Mathematics, Gdańsk University of Technology, Narutowicza 11/12, 80-233 Gdańsk, Poland

[4] BioTechMed Center, Gdańsk University of Technology, Poland

[5] Center for Space Biomedicine and Radiological Protection, Medical University of Gdansk, Gdansk, Poland

[6]Mathematics and Physics Department, School of Arts and Sciences, American University of Ras Al Khaimah, P. O. Box 10021, Ras Al-Khaimah, UAE

[*]The corresponding author:

hamid.berriche@aurak.ac.ae

Table S1: Vibrational energy levels trapped in the ground state $^{1}\Sigma_g^+$ of our homonuclear systems, using MRCI+Q level of theory with basis sets cc-pV5Z, aug-cc-pV5Z, and ECP28MWB_AVTZ for F_2, Cl_2, and Br_2, respectively.

	F_2		Cl_2		Br_2	
υ	E(υ) (cm^{-1})	E(υ)-E(υ-1) (cm^{-1})	E(υ) (cm^{-1})	E(υ)-E(υ-1) (cm^{-1})	E(υ) (cm^{-1})	E(υ)-E(υ-1) (cm^{-1})
0	-12369.151		-20005.101		-16049.704	
1	-11478.696	890.455	-19454.000	551.101	-15740.806	308.898
2	-10611.048	867.648	-18908.114	545.886	-15433.800	307.005
3	-9768.287	842.761	-18368.145	539.969	-15129.034	304.767
4	-8952.134	816.152	-17833.694	534.451	-14826.397	302.636
5	-8163.313	788.822	-17304.837	528.857	-14525.871	300.527
6	-7402.212	761.101	-16781.706	523.131	-14227.496	298.375
7	-6670.006	732.205	-16264.312	517.393	-13931.269	296.227
8	-5967.509	702.497	-15752.707	511.606	-13637.203	294.065
9	-5295.509	672.000	-15246.964	505.743	-13345.302	291.901
10	-4655.240	640.268	-14747.199	499.765	-13055.594	289.708
11	-4047.706	607.535	-14253.496	493.704	-12768.111	287.482
12	-3473.936	573.769	-13765.954	487.542	-12482.878	285.233
13	-2935.253	538.683	-13284.681	481.273	-12199.912	282.966
14	-2432.832	502.422	-12809.793	474.888	-11919.221	280.691
15	-1968.010	464.820	-12341.336	468.457	-11640.811	278.410
16	-1542.146	425.864	-11879.258	462.078	-11364.703	276.108
17	-1156.533	385.613	-11423.443	455.815	-11090.919	273.784
18	-812.340	344.193	-10973.793	449.649	-10819.480	271.439
19	-510.137	302.203	-10530.272	443.521	-10550.405	269.075
20	-249.285	260.852	-10092.894	437.378	-10283.709	266.695
21	-25.607	223.678	-9661.770	431.124	-10019.410	264.299
22			-9237.102	424.668	-9757.529	261.881
23			-8819.104	417.998	-9498.094	259.435
24			-8407.991	411.113	-9241.131	256.963
25			-8003.949	404.042	-8986.667	254.464
26			-7607.088	396.861	-8734.726	251.940
27			-7217.466	389.623	-8485.331	249.395
28			-6835.157	382.308	-8238.504	246.827
29			-6460.254	374.903	-7994.275	244.228
30			-6092.830	367.424	-7752.676	241.599
31			-5732.973	359.857	-7513.738	238.937
32			-5380.789	352.184	-7277.494	236.245
33			-5036.387	344.402	-7043.969	233.524
34			-4699.907	336.480	-6813.196	230.774
35			-4371.504	328.403	-6585.204	227.992
36			-4051.369	320.134	-6360.030	225.174
37			-3739.729	311.641	-6137.713	222.318
38			-3436.859	302.870	-5918.288	219.424
39			-3143.102	293.757	-5701.794	216.494
40			-2858.903	284.199	-5488.267	213.527
41			-2584.872	274.031	-5277.743	210.523
42			-2321.879	262.993	-5070.267	207.476

43			-2071.047	250.832	-4865.883	204.384
44			-1833.402	237.645	-4664.637	201.246
45			-1609.128	224.272	-4466.576	198.062
46			-1397.438	211.690	-4271.743	194.832
47			-1197.903	199.535	-4080.188	191.555
48			-1011.165	186.737	-3891.963	188.225
49			-837.916	173.249	-3707.124	184.839
50			-678.290	159.625	-3525.725	181.399
51			-532.433	145.857	-3347.821	177.904
52			-400.437	131.996	-3173.468	174.352
53			-281.765	118.672	-3002.727	170.741
54			-174.574	107.191	-2835.663	167.064
55			-74.559	100.015	-2672.341	163.323
56					-2512.826	159.514
57					-2357.191	155.635
58					-2205.508	151.683
59					-2057.856	147.653
60					-1914.311	143.544
61					-1774.955	139.356
62					-1639.869	135.085
63					-1509.142	130.728
64					-1382.863	126.278
65					-1261.128	121.735
66					-1144.035	117.093
67					-1031.686	112.349
68					-924.187	107.498
69					-821.649	102.539
70					-724.183	97.466
71					-631.910	92.273
72					-544.951	86.959
73					-463.432	81.519
74					-387.484	75.947
75					-317.237	70.247
76					-252.826	64.411
77					-194.386	58.439
78					-142.015	52.372
79					-95.126	46.888
80					-50.353	44.773
81					-2.374	47.979

Table S2: Vibrational energy levels trapped in the ground state $^2\Pi$ of our heteronuclear systems, using MRCI+Q level of theory with basis sets cc-pVQZ , aug-cc-pV6Z, and ECP28MWB_AVQZ/cc-pVQZ, for FO, ClO, and BrO, respectively.

	**FO**		**ClO**		**BrO**	
υ	E(υ) (cm^{-1})	E(υ)-E(υ-1) (cm^{-1})	E(υ) (cm^{-1})	E(υ)-E(υ-1) (cm^{-1})	E(υ) (cm^{-1})	E(υ)-E(υ-1) (cm^{-1})
0	-16836.735		-21452.773		-18750.825	
1	-15806.543	1030.192	-20622.308	830.465	-18050.447	700.377
2	-14798.152	1008.392	-19802.882	819.426	-17359.322	691.125
3	-13810.875	987.276	-18994.796	808.085	-16677.096	682.226

4	-12845.734	965.141	-18197.403	797.394	-16004.544	672.552
5	-11903.242	942.491	-17410.957	786.446	-15341.76	662.780
6	-10984.543	918.699	-16636.057	774.899	-14688.588	653.175
7	-10090.390	894.153	-15872.966	763.091	-14045.148	643.440
8	-9221.339	869.051	-15121.813	751.153	-13411.653	633.496
9	-8378.421	842.918	-14382.855	738.958	-12788.221	623.432
10	-7562.738	815.683	-13656.425	726.429	-12175.032	613.188
11	-6775.269	787.469	-12942.712	713.714	-11572.300	602.732
12	-6017.427	757.842	-12241.943	700.768	-10980.288	592.012
13	-5291.001	726.427	-11554.426	687.517	-10399.194	581.094
14	-4597.929	693.070	-10880.527	673.898	-9829.232	569.962
15	-3940.661	657.268	-10220.559	659.968	-9270.661	558.571
16	-3322.270	618.391	-9574.857	645.702	-8723.793	546.867
17	-2746.040	576.229	-8943.818	631.039	-8188.918	534.875
18	-2215.740	530.300	-8327.896	615.922	-7666.325	522.593
19	-1735.417	480.323	-7727.523	600.373	-7156.349	509.975
20	-1309.008	426.409	-7143.169	584.354	-6659.387	496.962
21	-940.374	368.633	-6575.394	567.775	-6175.839	483.547
22	-632.770	307.603	-6024.858	550.535	-5706.103	469.736
23	-388.695	244.075	-5492.291	532.567	-5250.638	455.465
24	-211.312	177.383	-4978.537	513.753	-4809.962	440.676
25	-97.160	114.151	-4484.574	493.964	-4384.598	425.363
26	-21.888	75.271	-4011.412	473.162	-3975.088	409.510
27			-3560.059	451.352	-3582.054	393.033
28			-3131.628	428.432	-3206.155	375.899
29			-2727.242	404.385	-2848.048	358.106
30			-2347.953	379.289	-2508.455	339.592
31			-1994.865	353.087	-2188.139	320.316
32			-1669.103	325.762	-1887.846	300.294
33			-1371.759	297.344	-1608.298	279.547
34			-1103.896	267.863	-1350.200	258.098
35			-866.499	237.397	-1114.345	235.855
36			-660.468	206.029	-900.953	213.392
37			-486.544	173.925	-707.471	193.482
38			-345.098	141.446	-530.049	177.422
39			-235.179	109.918	-367.706	162.343
40			-152.037	83.143	-220.231	147.475
41			-87.651	64.386	-87.818	132.412
42			-40.730	46.920		
43			-9.041	31.689		

Table S3: Spectroscopic constants of the ground state of F_2O using MRCI+Q/AVQZ for different Jacobi angles θ and bond lengths *r* and *R*.

r(Å)	R(Å)	$E_{min}(\mathrm{cm^{-1}})$	$\omega_e(\mathrm{cm^{-1}})$
**θ =10.2041°**			
1.6	2.070	-4556.33	1255.44
1.8	2.185	-9796.53	1352.05
2.0	2.301	-11018.13	1326.83
2.2	2.594	-14434.84	907.63

2.4	2.533	-11274.63	1184.83
2.6	2.643	-11432.41	1134.10
2.8	2.748	-11615.51	1044.44
**θ =23.4225°**			
1.6	2.465	-26254.72	571.70
1.8	2.067	-14072.53	1292.01
2.0	2.173	-14308.74	1247.61
2.2	2.277	-13623.04	1161.76
2.4	2.153	-14003.44	1630.17
2.6	2.472	-12519.36	957.43
2.8	2.559	-12295.76	882.49
**θ =36.7189°**			
1.6	1.820	-13185.63	1170.79
1.8	1.890	-15868.14	1242.02
2.0	1.974	-15565.91	1174.95
2.2	2.057	-14440.86	1072.80
2.4	2.133	-13395.39	954.08
2.6	2.198	-12629.41	802.59
2.8	2.245	-12102.18	719.83
**θ =50.0328°**			
1.6	1.641	-11594.08	1084.06
1.8	1.678	-14587.17	1230.46
2.0	1.744	-14414.79	1182.84
2.2	1.837	-12693.29	1144.65
2.4	1.741	-10689.99	631.06
2.6	1.721	-11106.84	594.32
2.8	1.685	-13905.05	521.00
**θ =63.3530°**			
1.6	1.611	-9105.58	679.29
1.8	1.341	-11012.93	824.33
2.0	1.284	-17883.07	813.39
2.2	1.210	-21447.18	721.03
2.4	1.098	-23552.25	596.91
2.6	0.920	-25193.64	442.29
2.8	0.745	-26049.44	431.63
**θ =76.6759°**			
1.6	2.263	-8182.68	119.71
1.8	1.071	-26097.51	1021.56
2.0	0.997	-33960.94	944.69
2.2	0.917	-36807.83	860.61
2.4	0.835	-36669.93	775.90
2.6	0.758	-34418.34	700.36
2.8	0.691	-30562.64	630.30
**θ =90.0000°**			
1.6	2.576	-16175.04	76.48
1.8	0.986	-33660.80	1079.71
2.0	0.919	-40569.10	1012.37
2.2	0.853	-41936.89	931.36
2.4	0.788	-40095.01	848.57
2.6	0.726	-36269.96	763.29

2.8	0.667	-31222.34	678.54
**θ =103.3241°**			
1.6	2.263	-8182.68	119.71
1.8	1.071	-26097.45	1021.56
2.0	0.997	-33960.94	944.69
2.2	0.917	-36803.27	860.61
2.4	0.835	-36669.93	775.90
2.6	0.758	-34418.34	700.36
2.8	0.691	-30562.64	630.30
**θ =116.6470°**			
1.6	1.611	-9105.58	679.29
1.8	1.341	-11012.93	824.33
2.0	1.284	-17883.07	813.39
2.2	1.210	-21447.18	721.03
2.4	1.098	-23552.25	596.91
2.6	0.920	-25193.62	442.29
2.8	0.745	-26049.44	431.63
**θ =129.9672°**			
1.6	1.641	-11594.08	1084.06
1.8	1.678	-14587.17	1230.46
2.0	1.744	-14414.79	1182.84
2.2	1.837	-12693.29	1144.65
2.4	1.741	-10689.99	631.04
2.6	1.720	-11106.35	594.94
2.8	1.685	-13905.06	521.00
**θ =143.2811°**			
1.6	1.820	-13185.63	1170.79
1.8	1.890	-15868.15	1242.02
2.0	1.971	-15565.91	1174.95
2.2	2.057	-14440.86	1072.80
2.4	2.133	-13395.39	954.08
2.6	2.198	-12629.42	802.58
2.8	2.245	-12102.18	719.83
**θ =156.5775°**			
1.6	2.465	-26254.72	571.70
1.8	2.067	-14072.53	1292.01
2.0	2.173	-14308.74	1247.61
2.2	2.277	-13623.04	1161.76
2.4	2.153	-14003.44	1630.17
2.6	2.472	-12519.64	958.02
2.8	2.559	-12294.18	879.83
**θ =169.7959°**			
1.6	2.070	-4555.45	1254.05
1.8	2.185	-9796.53	1352.05
2.0	2.301	-11018.35	1325.57
2.2	2.594	-14434.86	907.63
2.4	2.533	-11274.63	1184.83
2.6	2.643	-11434.62	1134.10
2.8	2.751	-11733.97	1176.96

Table S4: Spectroscopic constants of Cl_2O configuration using MRCI+Q/AVQZ for different Jacobi angles θ and bond lengths *r* and *R*.

$\boldsymbol{r}$(Å)	**R**(Å)	$\boldsymbol{E_{min}}(\mathbf{cm^{-1}})$	$\boldsymbol{\omega_e}(\mathbf{cm^{-1}})$
**θ =10.2041°**			
2.2	2.588	-24978.91	932.26
2.4	2.682	-25069.13	934.49
2.6	2.782	-23596.94	913.03
2.8	2.886	-21676.03	880.79
3.0	2.991	-19865.15	850.03
3.2	3.098	-18391.59	819.16
3.4	3.203	-17288.43	802.74
**θ =23.4225°**			
2.2	2.442	-32356.04	930.72
2.4	2.521	-31191.78	929.49
2.6	2.605	-28810.88	900.84
2.8	2.690	-26114.16	875.23
3.0	2.777	-23577.87	844.21
3.2	2.864	-21416.72	812.28
3.4	2.950	-19692.40	779.69
**θ =36.7189°**			
2.2	2.222	-35973.29	885.53
2.4	2.274	-33769.26	879.21
2.6	2.329	-30682.09	848.32
2.8	2.383	-27484.78	804.69
3.0	2.438	-24573.43	758.47
3.2	2.489	-22123.61	708.40
3.4	2.536	-20181.01	665.89
**θ =50.0328°**			
2.2	1.971	-34019.69	789.07
2.4	1.980	-31268.78	781.50
2.6	1.992	-28026.02	746.88
2.8	2.003	-24881.88	694.31
3.0	2.010	-22134.34	633.58
3.2	2.007	-19943.08	562.64
3.4	1.987	-18403.40	484.39
**θ =63.3530°**			
2.2	1.782	-24925.30	570.24
2.4	1.670	-22256.54	595.15
2.6	1.563	-22465.15	526.99
2.8	1.417	-24931.47	535.04
3.0	1.271	-27273.69	427.29
3.2	1.034	-29360.74	290.29
3.4	0.833	-30547.64	302.36
**θ =76.6759°**			
2.2	1.310	-18820.00	811.27
2.4	1.233	-29512.33	718.82
2.6	1.139	-35385.56	649.39
2.8	1.034	-38154.07	578.14
3.0	0.927	-38660.25	513.62

3.2	0.835	-37258.91	468.21
3.4	0.767	-34248.69	439.68
**θ =90.0000°**			
2.2	1.204	-28440.61	783.73
2.4	1.120	-37759.67	750.18
2.6	1.034	-42182.30	688.73
2.8	0.948	-43303.62	623.23
3.0	0.866	-42086.36	563.10
3.2	0.794	-39146.32	513.93
3.4	0.735	-34978.74	474.13
**θ =103.3241°**			
2.2	1.309	-18819.57	817.20
2.4	1.233	-29512.49	718.82
2.6	1.139	-35385.56	649.38
2.8	1.034	-38154.07	578.14
3.0	0.927	-38660.42	513.62
3.2	0.835	-37258.91	468.21
3.4	0.767	-34248.59	439.68
**θ =116.6470°**			
2.2	1.782	-24925.30	570.24
2.4	1.670	-22256.54	595.15
2.6	1.563	-22487.08	526.99
2.8	1.417	-24911.72	535.04
3.0	1.271	-27273.73	427.29
3.2	1.034	-29360.74	290.29
3.4	0.833	-30547.64	302.36
**θ =129.9672°**			
2.2	1.971	-34019.69	789.08
2.4	1.980	-31268.76	781.50
2.6	2.332	-30707.69	863.49
2.8	2.003	-24881.87	694.31
3.0	2.010	-22134.34	633.58
3.2	2.007	-19943.08	562.64
3.4	1.978	-18403.42	484.39
**θ =143.2811°**			
2.2	2.222	-35973.29	885.53
2.4	2.274	-33769.26	879.21
2.6	2.329	-30682.08	848.32
2.8	2.383	-27484.79	804.69
3.0	2.438	-24573.43	758.47
3.2	2.489	-22123.60	708.40
3.4	2.536	-20183.66	668.63
**θ =156.5775°**			
2.2	2.442	-32356.04	930.73
2.4	2.521	-31191.77	929.49
2.6	2.605	-28810.88	900.84
2.8	2.690	-26114.16	875.23
3.0	2.777	-23577.88	844.21
3.2	2.864	-21416.72	812.28
3.4	2.950	-19692.40	779.69

θ =169.7959°			
2.2	2.588	-24978.93	932.39
2.4	2.286	-25069.14	934.48
2.6	2.782	-23596.94	913.03
2.8	2.886	-21662.86	880.79
3.0	2.991	-19865.15	850.03
3.2	3.098	-18391.59	819.21
3.4	3.203	-17288.43	802.73

Table S5: Spectroscopic constants of Br_2O using MRCI+Q/AVQZ for different Jacobi angles θ and bond lengths *r* and *R*.

r(Å)	R(Å)	$E_{min}(\mathrm{cm^{-1}})$	$\omega_e(\mathrm{cm^{-1}})$
**θ =10.2041°**			
2.4	2.831	-24751.33	811.99
2.6	2.927	-25571.03	853.16
2.8	3.023	-24546.10	853.99
3.0	3.122	-22841.44	841.76
3.2	3.224	-21118.98	826.28
3.4	3.325	-19615.96	819.96
3.6	3.426	-18389.09	800.05
**θ =23.4225°**			
2.4	2.681	-31861.28	815.75
2.6	2.755	-31374.05	842.92
2.8	2.835	-29521.94	833.67
3.0	2.916	-27130.97	822.00
3.2	3.224	-21119.14	826.28
3.4	3.081	-22617.09	780.96
3.6	3.164	-20862.59	763.49
**θ =36.7189°**			
2.4	2.441	-35627.27	783.63
2.6	2.488	-34222.63	789.18
2.8	2.537	-31581.91	775.04
3.0	2.588	-28646.46	744.97
3.2	2.636	-25863.06	724.62
3.4	2.683	-23456.06	698.16
3.6	2.727	-21454.25	660.23
**θ =50.0328°**			
2.4	2.161	-34609.34	690.30
2.6	2.167	-32466.50	688.78
2.8	2.171	-29408.89	674.37
3.0	2.175	-26316.14	656.02
3.2	2.172	-23533.99	611.23
3.4	2.164	-21233.09	564.47
3.6	2.727	-21454.25	660.23
**θ =63.3530°**			
2.4	1.936	-27769.79	539.79
2.6	1.881	-24722.48	524.39
2.8	1.772	-21682.92	405.01

3.0	1.585	-22811.65	655.71
3.2	1.477	-24703.08	452.91
3.4	1.283	-26549.14	284.03
3.6	0.851	-28754.64	171.51
**θ =76.6759°**			
2.4	1.858	-17615.34	243.26
2.6	1.357	-27098.70	637.89
2.8	1.257	-32824.79	547.28
3.0	1.144	-35952.97	499.18
3.2	1.015	-37198.49	442.34
3.4	0.895	-36794.21	388.33
3.6	0.806	-34819.21	340.71
**θ =90.0000°**			
2.4	1.309	-27408.97	694.69
2.6	1.219	-36036.37	655.58
2.8	1.126	-36081.36	604.64
3.0	1.029	-42048.66	547.83
3.2	0.930	-41604.44	491.12
3.4	0.842	-39545.55	440.25
3.6	0.766	-36196.58	398.94
**θ =103.3241°**			
2.4	1.900	-17589.53	413.15
2.6	1.357	-27093.66	629.35
2.8	1.257	-32824.86	547.28
3.0	1.141	-35938.92	478.74
3.2	1.015	-37198.57	441.79
3.4	0.895	-36794.21	388.33
3.6	0.806	-34819.21	340.71
**θ =116.6470°**			
2.4	1.936	-27769.64	539.79
2.6	1.881	-24722.45	524.39
2.8	1.772	-21682.73	405.01
3.0	1.585	-22811.05	654.73
3.2	1.481	-24695.67	468.69
3.4	1.283	-26549.14	284.03
3.6	0.851	-28754.64	171.43
**θ =129.9672°**			
2.4	2.161	-34609.34	690.30
2.6	2.167	-32466.50	688.78
2.8	2.171	-29409.06	674.37
3.0	2.174	-26306.21	651.39
3.2	2.172	-23533.29	610.79
3.4	2.164	-21233.11	564.47
3.6	2.142	-19465.57	507.08
**θ =143.2811°**			
2.4	2.441	-35627.28	783.63
2.6	2.488	-34222.75	789.18
2.8	2.537	-31581.92	775.02
3.0	2.586	-28849.45	745.73
3.2	2.636	-25863.17	724.62

3.4	2.683	-23456.06	698.16
3.6	2.727	-21454.26	660.23
**θ =156.5775°**			
2.4	2.681	-31861.28	815.75
2.6	2.755	-31440.09	843.17
2.8	2.835	-29521.94	833.67
3.0	2.916	-27131.91	825.08
3.2	2.999	-24744.52	792.56
3.4	3.081	-22617.03	780.96
3.6	3.164	-20862.59	763.49
**θ =169.7959°**			
2.4	2.831	-24751.05	811.82
2.6	2.927	-25566.99	854.97
2.8	3.023	-24544.87	853.81
3.0	3.123	-22848.75	835.71
3.2	3.221	-21241.83	879.45
3.4	3.325	-19615.89	819.93
3.6	3.426	-18389.09	800.05

Table S6: Spectroscopic constants of $FO-F$ using MRCI+Q/AVQZ for different Jacobi angles θ and bond lengths *r* and *R*.

r(Å)	**R**(Å)	$E_{min}(\mathbf{cm^{-1}})$	$\omega_e(\mathbf{cm^{-1}})$
**θ =10.2041°**			
1.0	2.374	29314.95	487.12
1.2	2.448	-9710.18	427.09
1.4	2.434	-15680.11	385.61
1.6	2.265	-16315.86	642.59
1.8	2.310	-16742.09	833.49
**θ =23.4225°**			
1.0	2.172	20122.19	555.64
1.2	2.168	-19053.81	488.42
1.4	2.098	-27269.51	605.50
1.6	2.113	-27621.98	779.64
1.8	2.174	-25176.25	869.76
**θ =36.7189°**			
1.0	1.954	10959.94	583.39
1.2	1.900	-29480.51	634.31
1.4	1.902	-37468.91	760.49
1.6	1.933	-35555.57	827.92
1.8	1.978	-30754.97	842.59
**θ =50.0328°**			
1.0	1.790	4895.26	644.29
1.2	1.742	-35272.51	735.19
1.4	1.738	-41899.88	790.36
1.6	1.746	-38239.33	795.35
1.8	1.757	-31923.55	773.43
**θ =63.3530°**			
1.0	1.726	5323.05	650.27

1.2	1.664	-33360.07	705.89
1.4	1.633	-38889.59	736.54
1.6	1.613	-34266.46	722.77
1.8	1.591	-34266.46	686.84
**θ =76.6759°**			
1.0	1.757	11763.31	642.79
1.2	1.707	-24447.62	608.66
1.4	1.656	-28747.82	617.17
1.6	1.618	-23629.81	620.04
1.8	1.582	-16268.96	597.00
**θ =90.0000°**			
1.0	1.845	19904.59	595.15
1.2	1.864	-13713.68	508.55
1.4	1.871	-16680.29	427.11
1.6	1.833	-10972.89	423.68
1.8	1.833	-2302.31	494.87
**θ =103.3241°**			
1.0	1.890	23507.36	565.07
1.2	1.926	-8916.21	447.67
1.4	2.181	-10523.34	193.65
1.6	Repulsive		0
1.8	1.488	2397.82	567.96
**θ =116.6470°**			
1.0	1.900	21668.19	578.47
1.2	1.972	-12174.13	522.02
1.4	2.337	-13614.57	287.51
1.6	1.627	-4327.89	669.96
1.8	1.598	-4281.21	733.17
**θ =129.9672°**			
1.0	1.963	20735.53	595.57
1.2	2.066	-13392.13	516.79
1.4	2.465	-13782.99	235.03
1.6	1.768	-6958.51	718.13
1.8	1.775	-6294.34	780.76
**θ =143.2811°**			
1.0	2.064	22738.83	578.21
1.2	2.177	-12321.56	507.73
1.4	2.587	-13160.66	204.10
1.6	1.929	-6222.04	717.22
1.8	1.960	-5899.11	818.22
**θ =156.5775°**			
1.0	2.187	27228.36	524.95
1.2	2.295	-9243.66	457.40
1.4	Repulsive	0	0
1.6	2.067	-3354.36	681.37
1.8	2.110	-4225.43	825.69
**θ =169.7959°**			
1.0	2.330	32590.35	473.51
1.2	2.438	-4593.27	334.75
1.4	Repulsive	0	0

1.6	2.145	1010.32	639.42
1.8	2.197	-1928.12	865.07

Table S7: Spectroscopic constants of $ClO - Cl$ using MRCI+Q/AVQZ for different Jacobi angles θ and bond lengths *r* and *R*.

$\boldsymbol{r}$(Å)	**R**(Å)	$\boldsymbol{E_{min}}(\mathbf{cm^{-1}})$	$\boldsymbol{\omega_e}(\mathbf{cm^{-1}})$
**θ =10.2041°**			
1.3	3.143	-3370.24	247.46
1.5	2.635	-23493.25	574.71
1.7	2.776	-26563.90	555.76
1.9	2.901	-24522.76	532.97
2.1	2.993	-23426.95	570.48
**θ =23.4225°**			
1.3	2.804	-9113.19	196.62
1.5	2.559	-32521.30	492.06
1.7	2.651	-36848.04	533.41
1.9	2.737	-35112.49	547.89
2.1	2.816	-32201.85	558.94
**θ =36.7189°**			
1.3	2.469	-14363.71	257.80
1.5	2.400	-38326.04	473.05
1.7	2.448	-42777.41	519.04
1.9	2.497	-40356.89	516.64
2.1	2.538	-36093.38	509.71
**θ =50.0328°**			
1.3	2.426	-14374.29	281.58
1.5	2.259	-37385.28	422.85
1.7	2.256	-41659.45	469.32
1.9	2.269	-38534.39	465.49
2.1	2.275	-33097.66	444.41
**θ =63.3530°**			
1.3	2.463	-10444.72	307.81
1.5	2.305	-29341.87	288.86
1.7	2.199	-32489.61	365.88
1.9	2.180	-28853.22	386.01
2.1	2.168	-22580.35	401.12
**θ =76.6759°**			
1.3	2.468	-8591.00	304.75
1.5	2.505	-22391.84	258.02
1.7	2.616	-20651.39	506.31
1.9	2.510	-14439.44	184.32
2.1	1.606	-8971.02	528.66
**θ =90.0000°**			
1.3	2.333	-14958.99	327.42
1.5	2.374	-25984.78	266.21
1.7	2.106	-21016.09	291.48
1.9	1.999	-17293.89	436.49
2.1	1.964	-14013.15	460.38

θ =103.3241°			
1.3	2.247	-21762.06	360.25
1.5	2.226	-32564.42	317.51
1.7	2.117	-28624.02	390.85
1.9	2.666	-23117.29	467.49
2.1	2.051	-18462.80	485.43
**θ =116.6470°**			
1.3	2.266	-25280.44	385.69
1.5	2.262	-36130.96	365.32
1.7	2.213	-31778.79	412.08
1.9	2.186	-25231.07	476.26
2.1	2.190	-19854.53	494.24
**θ =129.9672°**			
1.3	2.348	-24969.52	385.87
1.5	2.363	-36173.27	369.33
1.7	2.335	-31785.29	410.48
1.9	2.320	-24961.44	476.59
2.1	2.338	-19535.47	501.97
**θ =143.2811°**			
1.3	2.467	-21527.42	366.26
1.5	2.492	-33409.41	347.42
1.7	2.466	-29333.94	382.78
1.9	2.448	-22785.93	467.43
2.1	2.473	-17889.22	502.45
**θ =156.5775°**			
1.3	2.604	-16120.87	342.17
1.5	2.639	-28748.82	325.45
1.7	2.613	-24931.89	322.07
1.9	2.561	-18893.57	440.20
2.1	2.584	-15107.72	493.02
**θ =169.7959°**			
1.3	2.724	-10299.05	322.09
1.5	2.766	-23294.47	307.69
1.7	2.786	-19453.30	273.87
1.9	2.656	-13509.81	389.51
2.1	2.658	-11527.01	481.18

Table S8: Spectroscopic constants of $BrO - Br$ using MRCI+Q/AVQZ for different Jacobi angles θ and bond lengths r and R.

r(Å)	R(Å)	$E_{min}(\mathrm{cm^{-1}})$	$\omega_e(\mathrm{cm^{-1}})$
**θ =10.2041°**			
1.4	2.901	329.33	368.96
1.6	3.056	-25805.12	395.32
1.8	3.221	-30243.53	385.59
2.0	3.380	-27901.91	375.44
2.2	3.515	-25192.52	378.32
**θ =23.4225°**			
1.4	2.865	-5681.31	232.35

1.6	2.940	-32635.09	337.88
1.8	3.057	-38385.50	355.38
2.0	3.170	-36905.39	358.64
2.2	3.273	-33630.22	360.22
**θ =36.7189°**			
1.4	2.767	-8561.79	183.76
1.6	2.734	-35887.03	305.53
1.8	2.792	-42054.06	323.96
2.0	2.853	-40319.65	323.22
2.2	2.905	-36107.64	309.59
**θ =50.0328°**			
1.4	2.793	-6342.41	176.78
1.6	2.584	-31669.48	235.16
1.8	2.569	-37627.37	274.18
2.0	2.587	-35325.68	273.09
2.2	2.606	-29984.05	254.73
**θ =63.3530°**			
1.4	2.796	-2778.63	180.27
1.6	2.785	-23064.89	168.05
1.8	2.635	-25827.06	163.68
2.0	2.572	-22732.50	189.84
2.2	2.671	-14385.36	184.79
**θ =76.6759°**			
1.4	2.637	-6550.84	197.44
1.6	2.680	-22916.67	161.34
1.8	2.363	-20994.81	202.88
2.0	2.274	-18351.06	278.97
2.2	2.246	-14957.17	290.09
**θ =90.0000°**			
1.4	2.478	-13733.16	227.94
1.6	2.439	-29993.88	212.06
1.8	2.338	-28836.72	252.67
2.0	2.284	-24072.97	294.59
2.2	2.266	-19212.92	308.78
**θ =103.3241°**			
1.4	2.449	-18216.02	251.82
1.6	2.430	-34294.95	244.19
1.8	2.380	-32469.78	267.39
2.0	2.345	-26487.61	300.66
2.2	2.337	-20736.81	313.27
**θ =116.6470°**			
1.4	2.483	-19415.85	257.59
1.6	2.479	-35519.06	251.89
1.8	2.449	-33412.07	269.08
2.0	2.422	-26901.49	298.29
2.2	2.419	-20826.20	313.25
**θ =129.9672°**			
1.4	2.551	-17891.70	251.45
1.6	2.554	-34365.73	246.27
1.8	2.530	-32387.18	259.22

2.0	2.502	-25825.05	291.33
2.2	2.501	-19845.49	309.35
**θ =143.2811°**			
1.4	2.641	-14327.49	236.44
1.6	2.645	-31401.00	231.67
1.8	2.619	-29773.91	240.01
2.0	2.580	-23425.81	281.11
2.2	2.577	-17899.69	305.96
**θ =156.5775°**			
1.4	2.746	-9528.73	219.13
1.6	2.749	-27238.39	214.69
1.8	2.722	-25929.43	214.32
2.0	2.656	-19814.81	261.97
2.2	2.641	-15094.22	297.59
**θ =169.7959°**			
1.4	2.842	-4591.99	203.84
1.6	2.842	-22695.79	201.44
1.8	2.839	-21450.33	191.52
2.0	2.734	-15167.79	232.38
2.2	2.688	-11643.19	284.47
